# Tailoring bifunctional hybrid organic–inorganic nanoadsorbents by the choice of functional layer composition probed by adsorption of Cu^2+^ ions

**DOI:** 10.3762/bjnano.8.36

**Published:** 2017-02-02

**Authors:** Veronika V Tomina, Inna V Melnyk, Yuriy L Zub, Aivaras Kareiva, Miroslava Vaclavikova, Gulaim A Seisenbaeva, Vadim G Kessler

**Affiliations:** 1 Chuiko Institute of Surface Chemistry of NASU, 17, Generala Naumova Str., Kyiv 03164, Ukraine; 2 Institute of Geotechnics SAS, 45, Watsonova, Kosice 04001, Slovak Republic; 3 Department of Inorganic Chemistry, Vilnius University, 24, Naugarduko Str., Vilnius LT-03225, Lithuania,; 4 Department of Chemistry and Biotechnology, Swedish University of Agricultural Sciences, 8, Almas allé, Uppsala 75007, Sweden

**Keywords:** copper(II) ions, methyl groups, N- and F-containing functional groups, silica nanospheres, sol–gel processes, sorption, surface chemistry

## Abstract

Spherical silica particles with bifunctional (≡Si(CH_2_)_3_NH_2_/≡SiCH_3_, ≡Si(CH_2_)_3_NH_2_/≡Si(CH_2_)_2_(CF_2_)_5_CF_3_) surface layers were produced by a one-step approach using a modified Stöber method in three-component alkoxysilane systems, resulting in greatly increased contents of functional components. The content of functional groups and thermal stability of the surface layers were analyzed by diffuse reflectance infrared Fourier transform (DRIFT) spectroscopy, and ^13^C and ^29^Si solid-state NMR spectroscopy revealing their composition and organization. The fine chemical structure of the surface in the produced hybrid adsorbent particles and the ligand distribution were further investigated by electron paramagnetic resonance (EPR) and electron spectroscopy of diffuse reflectance (ESDR) spectroscopy using Cu^2+^ ion coordination as a probe. The composition and structure of the emerging surface complexes were determined and used to provide an insight into the molecular structure of the surfaces. It was demonstrated that the introduction of short hydrophobic (methyl) groups improves the kinetic characteristics of the samples during the sorption of copper(II) ions and promotes fixation of aminopropyl groups on the surface of silica microspheres. The introduction of long hydrophobic (perfluoroctyl) groups changes the nature of the surface, where they are arranged in alternately hydrophobic/hydrophilic patches. This makes the aminopropyl groups huddled and less active in the sorption of metal cations. The size and aggregation/morphology of obtained particles was optimized controlling the synthesis conditions, such as concentrations of reactants, basicity of the medium, and the process temperature.

## Introduction

Materials with bi- or multifunctional surface layers have been of considerable interest in the recent years [1–11]. Such materials possess, undoubtedly, certain advantages over the materials with monofunctional coatings. For example, multifunctional drug-delivery agents may have functionalities ensuring their transportation to the desired target after introduction in the human body, whereas the functionalities of a different nature exert their, e.g., therapeutic effect [12]. Moreover, the creation of a multifunctional surface layer is an instrument for the subtle tuning of the sorption properties of materials by varying the nature, as well as the ratio of functional groups in the surface layer. Furthermore, combining hydrophobic and hydrophilic groups, reveals opportunities for creating, for example, the “pockets” with hydrophobic walls and a hydrophilic center (sorption or catalytic) [13], as well as the possibility of influencing the hydrophobicity of the surface layer.

The most suitable objects for such application are apparently hybrid organic–inorganic materials bearing surface-anchored functional groups. The methodology for their synthesis has reached a high-level, opening for broad practical application [14]. It is noteworthy that, as early as in the later part of the 20th century, Slinyakova et al. [15] drew attention to the extended possibilities of constructing new hybrid adsorbents systematizing data on polysiloxane xerogels containing hydrophobic surface layers, (O_3/2_SiR')*_x_* and [(SiO_2_)*_y_*(O_3/2_SiR')*_z_*], R' = –CH_3_, –C_2_H_5_, –CH=CH_2_, –C_6_H_5_. The papers describing the production of polysiloxane xerogels with complexing groups using one- [16] and two-component [17–19] (considering alkoxysilanes) systems brought about the understanding of such opportunities, and in [20] the results dealing with the synthesis, structure, and properties of polysiloxane xerogels with bifunctional surface layers were summarized. In the beginning of the 21st century, the studies of synthesis of mesoporous silicas functionalized with various groups emerged, [21–22] applying the well-known template method. Mann et al. were first to report it for the synthesis of hybrid organic–inorganic materials bearing functional groups of different nature [23–26]. Finally, there have been published papers describing the synthesis of non-porous silica nano- and sub-microspheres with functional groups in the surface layer [27–28]. The suggested technique was based on the Stöber method [29]. These silica spheres, because of their excellent properties, such as adjustable size and surface layer composition, along with their chemical stability and biocompatibility, are promising materials for the application in a wide range of areas (chromatography, controlled drug delivery, bioseparation, chemo- and biosensors, biocatalysis). Naturally, there arose a question about the possibility of synthesis for such particles with bi- and multi-functional surface layers.

The synthesis of functionalized silica nano- and sub-microspheres is achieved usually via two commonly used techniques. The first is based on the surface modification of Stöber silica spheres using trialkoxy silanes, (RO)_3_SiR'. The second technique applies a one-step synthesis, where the network-forming agent (usually tetraethoxysilane, TEOS) and the functionalizing agent, (RO)_3_SiR' are introduced simultaneously or sequentially (in a given order) in the reaction medium. However, already the very first reports showed that, despite their simplicity, both approaches were dependent on a multitude of not fully understood factors affecting the shape and size of the particles, the content of functional groups, hydrolytic stability of the surface layer, and even the final product yield.

The amino group is one of the most desired functions on surfaces. It is capable both to bind directly to metal cations and is also useful in further grafting of new functions via organic condensation reactions [30]. In the studies examining one-step preparations of nanoparticles bearing 3-aminopropyl groups it was shown that heating a suspension in DMF at 100 °C for 24 h did not increase the content of surface amino groups, but improved the stability of the surface layer [28]. It was shown [27] that the introduction of 3-aminopropyltriethoxysilane (APTES) to TEOS sol in ethanol in a molar ratio of 1:1 yielded nanoparticles with an average diameter around 66 nm, but their surface layer contained almost no amino groups. At a TEOS/APTES molar ratio of 3:1, the surface layer did contain amino groups (1.56 mmol/g), but the particle size decreases to about 9 nm. When the order of components introduction was changed, the content of functional groups increased to 3.2 mmol/g. However, according to ^29^Si NMR spectroscopy, the content of Q^4^ structural units decreased. This might indicate lower degree of cross-linking in the polysiloxane networks in the particles.

The most broadly used technique for the preparation of aminoalkyl functionalized silica has been, however, the surface modification of pre-produced nanoparticles with trialkoxy silanes. For example, in [31] APTES or phenуltriethoxysilane were used as modifiers. However, the amount of modifiers was negligible (4.79 × 10^−4^ mol), and the process time was as long as 19 h. The size of the particles bearing APTES was 150 nm (while the size of the initial TEOS particles was 149 nm). The moment when the second silane is added plays an important role. It was demonstrated that the availability of ligands on the surface could be affected by the time of addition for such functions as amino, monocarboxylate, ethylenediaminetriacetic acid, and dihydroimidazole-terminated ones [32]. The best surface availability of organofunctional groups was achieved for the amino-terminated ligands when the organosilane was added 30 min after start of the particle growth, and for the carboxylate-terminated ones the optimal addition time was 5.5 h after particle growth initialization. No efficient one-step approaches to dual functional particles bearing an aminopropyl group have been reported so far to the best of our knowledge.

The major interest in the studies of bifunctional particles bearing an amino function lies in achieving control over its stability to hydrolysis and especially over its availability and chemical reactivity. In monofunctional layers it is often involved in direct hydrogen bonding to the Si–OH groups on the surface, which decreases its chemical availability and can cause hydrolytic elimination of the siloxane fragment bearing this group [33–35]. The mechanisms of stabilization and the relation to the activity in adsorption of copper(II) cations has been more recently discussed in the works of Soler-Illia and co-workers [36–37]. Using DFT calculations it was demonstrated that the protonated amino groups are irreversibly transmitting a proton to the Si–O^−^ groups on the surface with subsequent breaking of the Si–O–Si bridges and potential release of silane species [36]. Apparently, the complexation with Cu(II) ions is additionally catalyzing this process, resulting in the loss of amino functions [37]. It was demonstrated that stability of the surface layer could be improved by gentle thermal treatment, leading to additional condensation of the –OH groups and elimination of the surface Si–O^−^ groups [37]. This transformation is, however, difficult to control. In the present work we propose to use a principally different strategy for stabilization of the surface layer inserting instead different hydrolytically stable hydrophobic functions, hindering the formation of H-bonds. We have applied two types of such groups, namely small ones with considerably reduced capacity to van der Waals bonding such as methyl groups, and rather large and long ones with potentially strong van der Waals bonding such as fluorinated groups.

We also aimed to test the availability and reactivity of the amino function via Cu^2+^-cation adsorption as a probe, using electron paramagnetic resonance (EPR) and electron spectroscopy of diffuse reflectance (ESDR) spectroscopy. This approach permits also to identify the nature of the surface complexes and thus probe the arrangement of the amino groups.

An effort has also been made to summarize the effects of factors influencing the size and aggregation of the produced particles.

## Results

We synthesized samples with aminopropyl groups, aminopropyl and methyl groups, aminopropyl and fluorine-containing groups. The samples, bearing only the aminopropyl groups were denoted as **N**, those bearing both aminopropyl and methyl groups as **NM** and those, bearing the fluorinated along with aminopropyl groups as **NF**. A detailed description is provided below in the Experimental section.

It is known that the temperature influences the process of hydrolysis and condensation of silanes, resulting in the formation of oligomers of different length. Therefore, the size of the silica particles depends on the synthesis temperature [38]. The particles with bifunctional surface layers at a TEOS/APTES/MTES (methyl triethoxysilane) ratio of 3:0.5:0.5 were synthesized at different temperatures (samples **NM**, **NMh** and **NMi**). Also bifunctional samples with amino and perfluorooctyl groups in the surface layer at various TEOS/APTES/PFES ratios (**NF1**–**NF4**) were synthesized. During synthesis APTES was first added to the ethanol–water–ammonium solution, and then the mixture of alkoxysilanes with different TEOS/PFES ratios was introduced.

**Figure 1:**
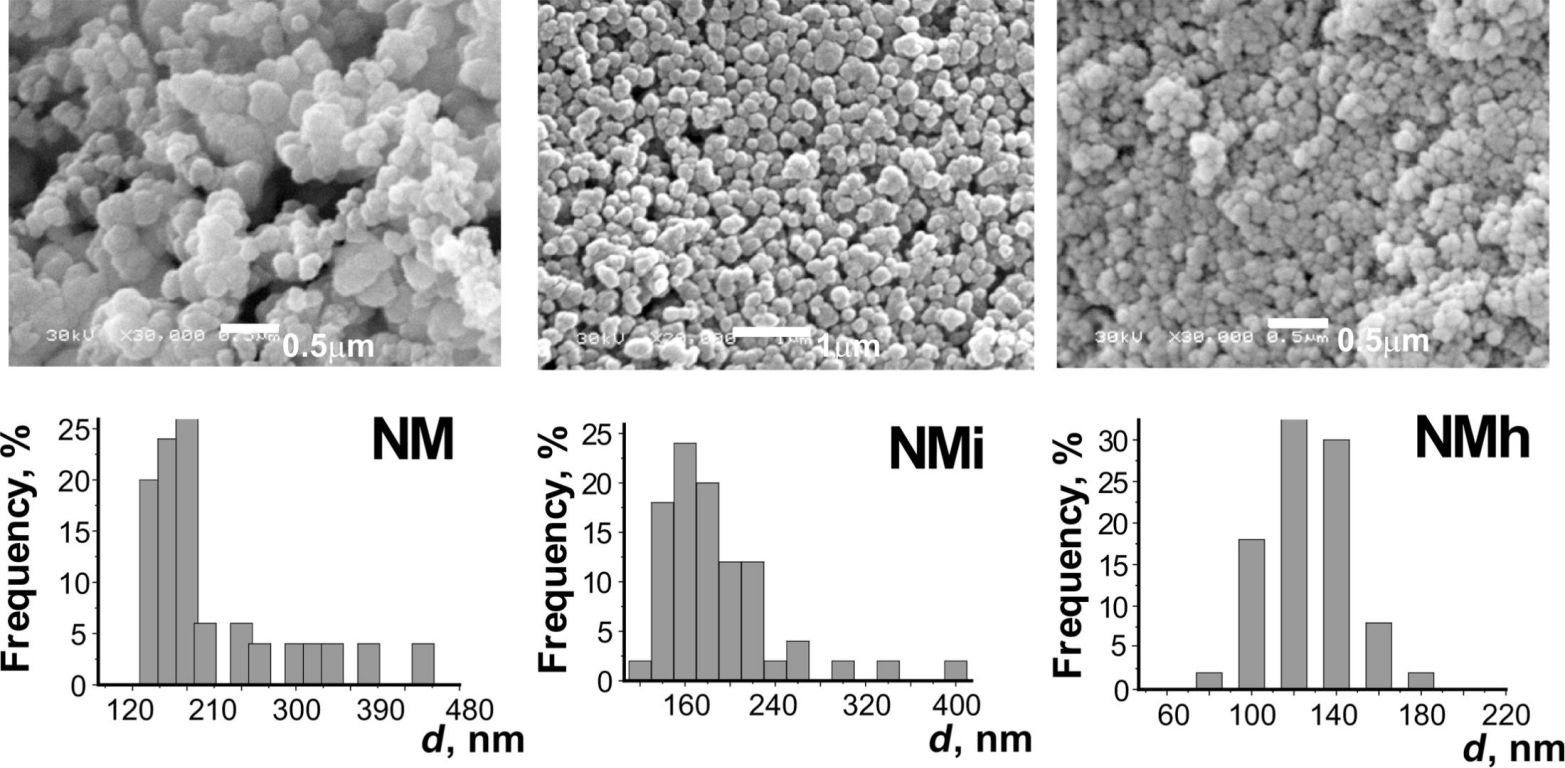
SEM images and particle size distribution curves for amino/methyl-containing samples **NM**, **NMi**, **NMh**.

**Figure 2:**
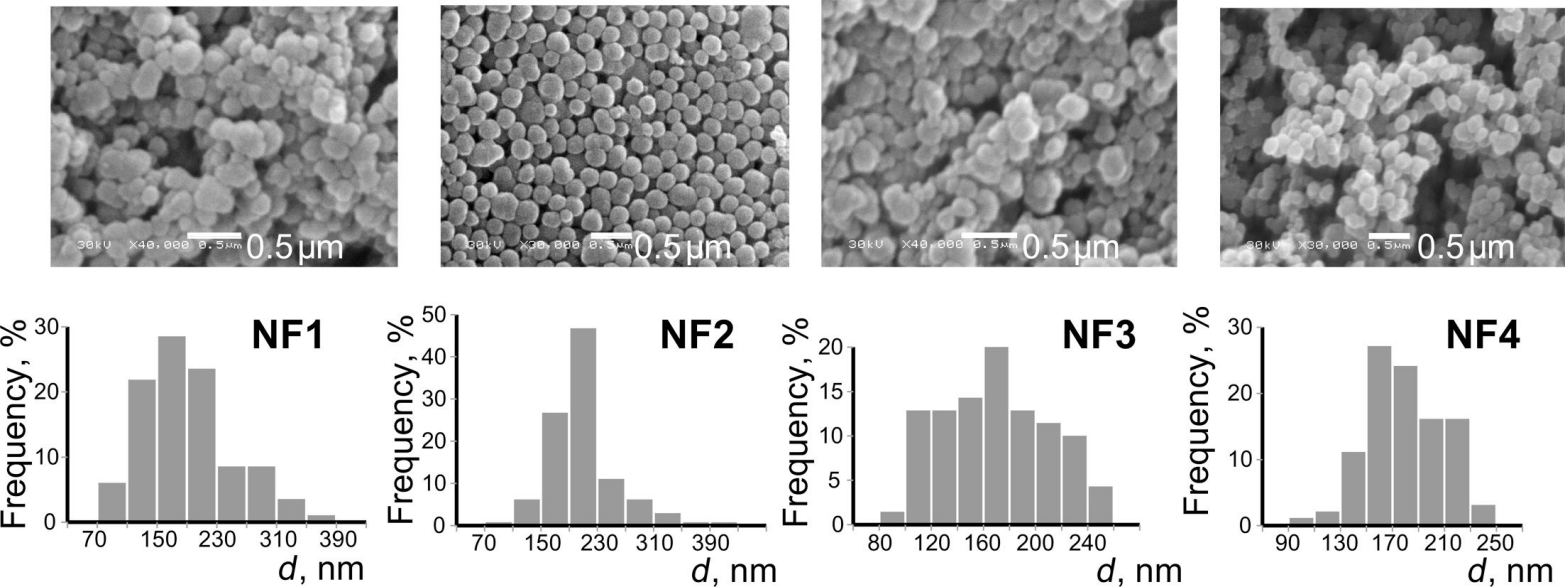
SEM images of amino-/fluorine-containing samples.

Morphology and particle size distribution of bifunctional silica samples were examined using SEM (Figure 1 and Figure 2). It is important to note that for the amino/methyl samples no direct link between the temperature and the particle size could be observed (contrary to aminosilica samples (Table S1, Supporting Information File 1). Lowering the temperature did not lead to a particle size change but the morphology of the particles was improved. At all temperatures, the sizes of the bifunctional amino/methyl particles that formed were smaller compared to monofunctional amino samples (Figure 1, Table S1, Supporting Information File 1). The nanoparticles with bifunctional amino-/fluorine-containing surface layer are not uniform in size (Figure 2), but their shape is close to spherical.

The particle size and morphological features were also reflected in the measured active surface area (see Table S1, Figure S1, Supporting Information File 1). The values were the highest for the bifunctional particles bearing methyl groups. The dominating feature of the monofunctional fluoroalkyl-substituted particles was that with increasing surface coverage they became more smooth and uniform in size (see Figure S3, Supporting Information File 1), which led to a loss of surface area.

The presence of target functional groups in the surface layers of the particles was confirmed by IR spectroscopy (diffuse reflectance infrared Fourier transform, DRIFT, for details see Figure S6, Supporting Information File 1) and their structure was analyzed by solid-state NMR.

**Figure 3:**
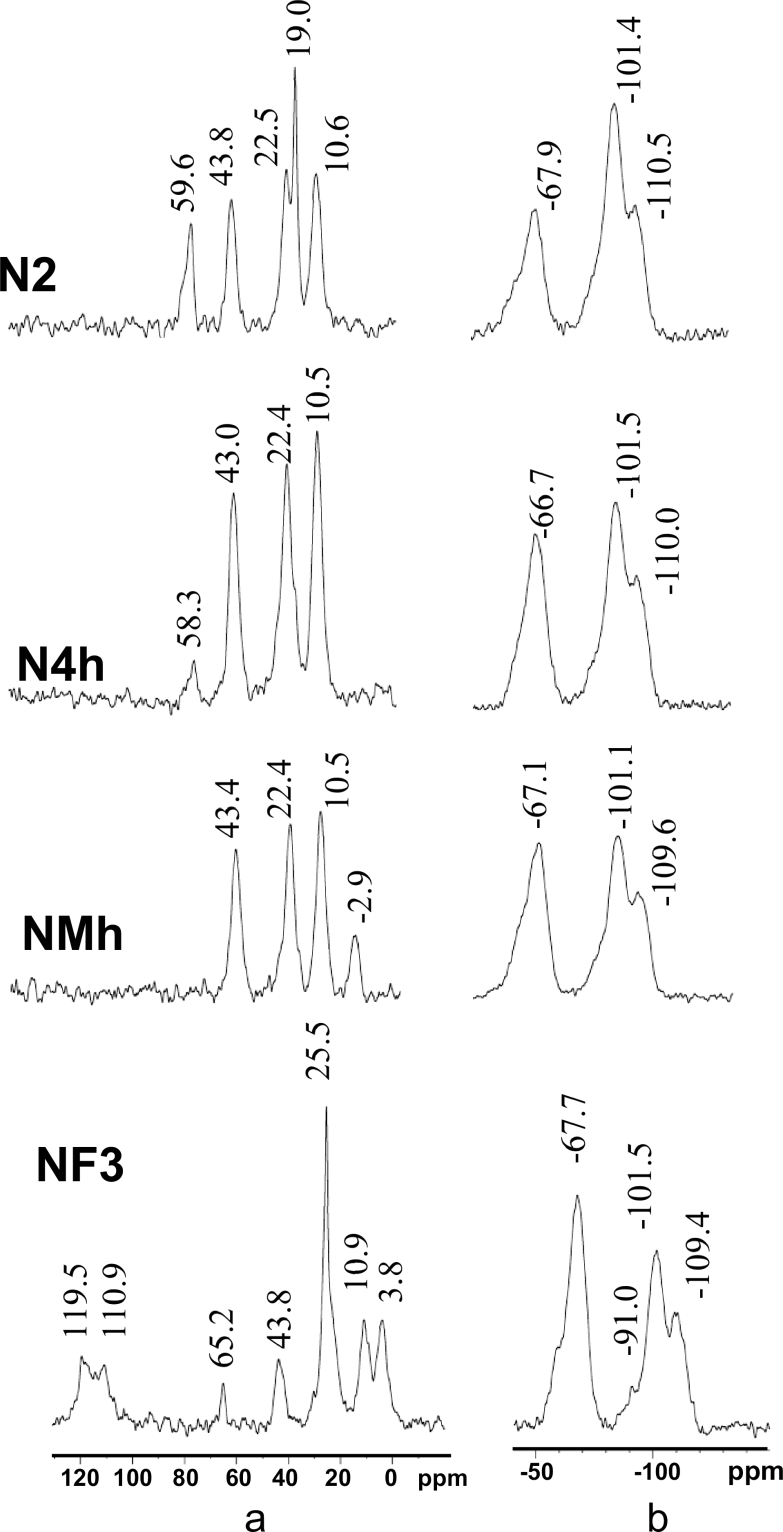
^13^C (a) and ^29^Si (b) CP/MAS NMR spectra of the synthesized samples.

All the ^13^C CP/MAS NMR spectra (Figure 3, Figure S2 and assignment in Tables S2 and S3, Supporting Information File 1) contained signals from three carbon atoms of the aminopropyl chain of APTES [27]. In addition, the spectra of some samples obtained at lower temperatures showed signals from carbon atoms of residual ethoxysilyl groups absent in the spectra of samples obtained at 50 °C.

In the case of the bifunctional samples (Figure 3), the ^13^C CP/MAS NMR spectrum of **NMh** also exhibits the signal of the carbon atom from methylsilyl group and the spectrum of **NF3** contains a group of signals in the region of 108–120 ppm characteristic of carbon atoms from the perfluoric chain (–(CF_2_)_5_CF_3_) [39]. The signal with the highest frequency (δ = 119.5 ppm) apparently corresponds to the carbon atom from CF_3_, and a shoulder at 108 ppm corresponds to the carbon atom from the CF_2_ group adjacent to CF_3_. The overlapping peaks at 110–113 ppm apparently belong to other CF_2_ carbon atoms. Two more signals in ^13^C CP/MAS NMR spectrum of **NF3** are due to the presence of perfluorooctyl groups of PFES. The peak at δ = 65.2 ppm originates from CH_2_ carbon adjacent to CF_2_ and the peak at δ = 3.8 ppm from the first carbon atom of the perfluorooctyl group bound to the silicon atom. Thus, the synthesized particles contain functional groups introduced with trialkoxy silanes during their syntheses.

The ^29^Si CP/MAS NMR spectra of the samples shown in Figure 3 are similar and contain two groups of signals. The first one (in the region from −110 to −90 ppm) indicates the presence of structural units Q^4^, Q^3^, and Q^2^. The signal with a chemical shift of −109 to −110 ppm is typical for silicon atom in a polysiloxane network Si(O_0.5_)_4_ (structural units of Q^4^ type). The signal at δ = −101 ppm refers to Q^3^ units, namely, the silicon atoms bound to terminal silanol groups Si(O_0.5_)_3_ОH or to unhydrolyzed ethoxy groups Si(O_0.5_)_3_ОC_2_H_5_. The weak signal at about −91 ppm, which is distinct for the sample **NF3** (for other samples it features a weak shoulder, see Figure 3), indicates the presence of Q^2^ structural units (Si(O_0.5_)_2_(ОR)_2_, where R = H or C_2_H_5_). The second group of signals (at about −68 ppm with a pronounced shoulder at −59 ppm, see Figure 3) belongs to T^3^ and T^2^ structural units, respectively (≡SiR’ and ≡Si(OR)R’, where R’ is a functional group –(CH_2_)_2_(CF_2_)_5_CF_3_ or –(CH_2_)_3_NH_2_) [40–41].

The contents of the functional groups present in mono- and bifunctional samples were quantified using thermal analysis (Figure S7 and Table S1, Supporting Information File 1), acid–base titration, elemental analysis and EDXS analysis (Table S4, Figure S4, Supporting Information File 1). According to these data, the content of amino groups in monofunctional silica particles is in the range of 0.5–2.0 mmol/g (at a ratio of TEOS/APTES = 3:1), which is about half of what is expected from the ratio of reacting alkoxysilanes. For the bifunctional samples with methyl groups we observed an amount of amino functional groups of 1.8–2.0 mmol/g at the ratio of TEOS/APTES/MTES = 3:0.5:0.5 (i.e. at half the applied amount of APTES). The content of methyl groups is about a third of what is expected from the ratio of silanes.

The content of amino and perfluorooctyl groups in bifunctional fluorinated samples (according to elemental analysis) generally agree with the theoretically calculated values. However, according to acid–base titration data not all amino groups are available for H^+^ sorption.

**Figure 4:**
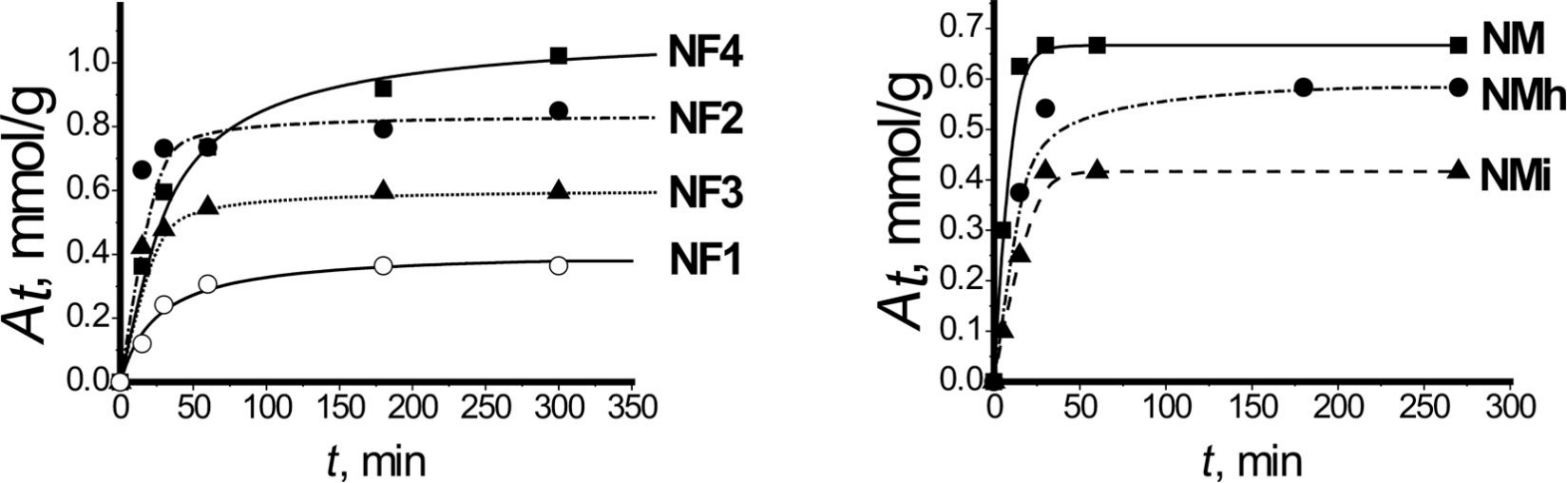
Pseudo-second-order kinetic curves for the sorption of copper(II) ions by the synthesized samples (the continuous lines are showing the fit of the model for each case with experimental points indicated by geometrical symbols).

**Figure 5:**
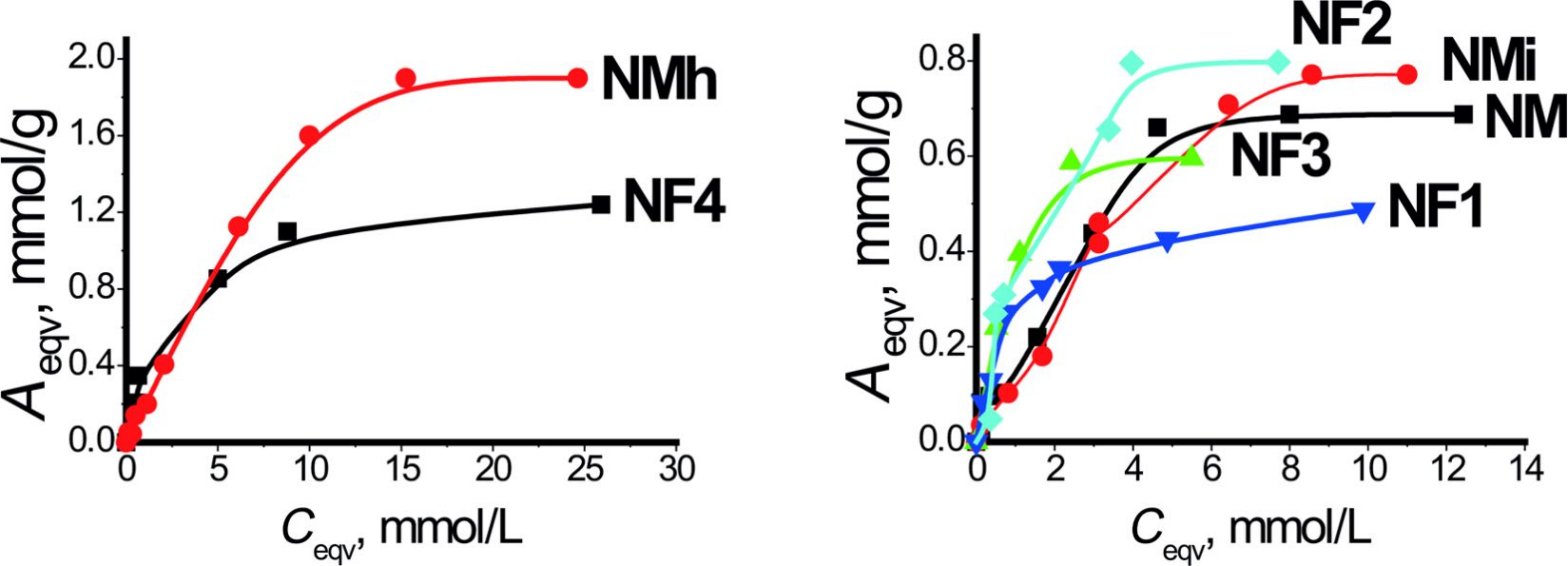
Copper(II) ion adsorption isotherms at 28 °C.

The intrinsic structure and reactivity of the aminopropyl groups were investigated using Cu^2+^ adsorption. In the view that EPR spectra of the copper(II) complexes provide direct insight into the coordination of these cations, they were thus revealing the spatial arrangement of the ligands. The kinetic studies were performed first to estimate the equilibrium time (see kinetic sorption parameters obtained using pseudo-first and pseudo-second-order models for metal ions sorption in Table S5, Supporting Information File 1). According to the presented data, the kinetic curves for the samples fit the pseudo-second-order model (Figure 4). The fastest is the reaction on amino/methyl bifunctional samples, where the equilibrium is reached within 30 min. Thus, based on the kinetic curves, specific times for the adsorption of copper(II) ions were chosen for each sample. The adsorption isotherms are presented in Figure 5.

**Figure 6:**
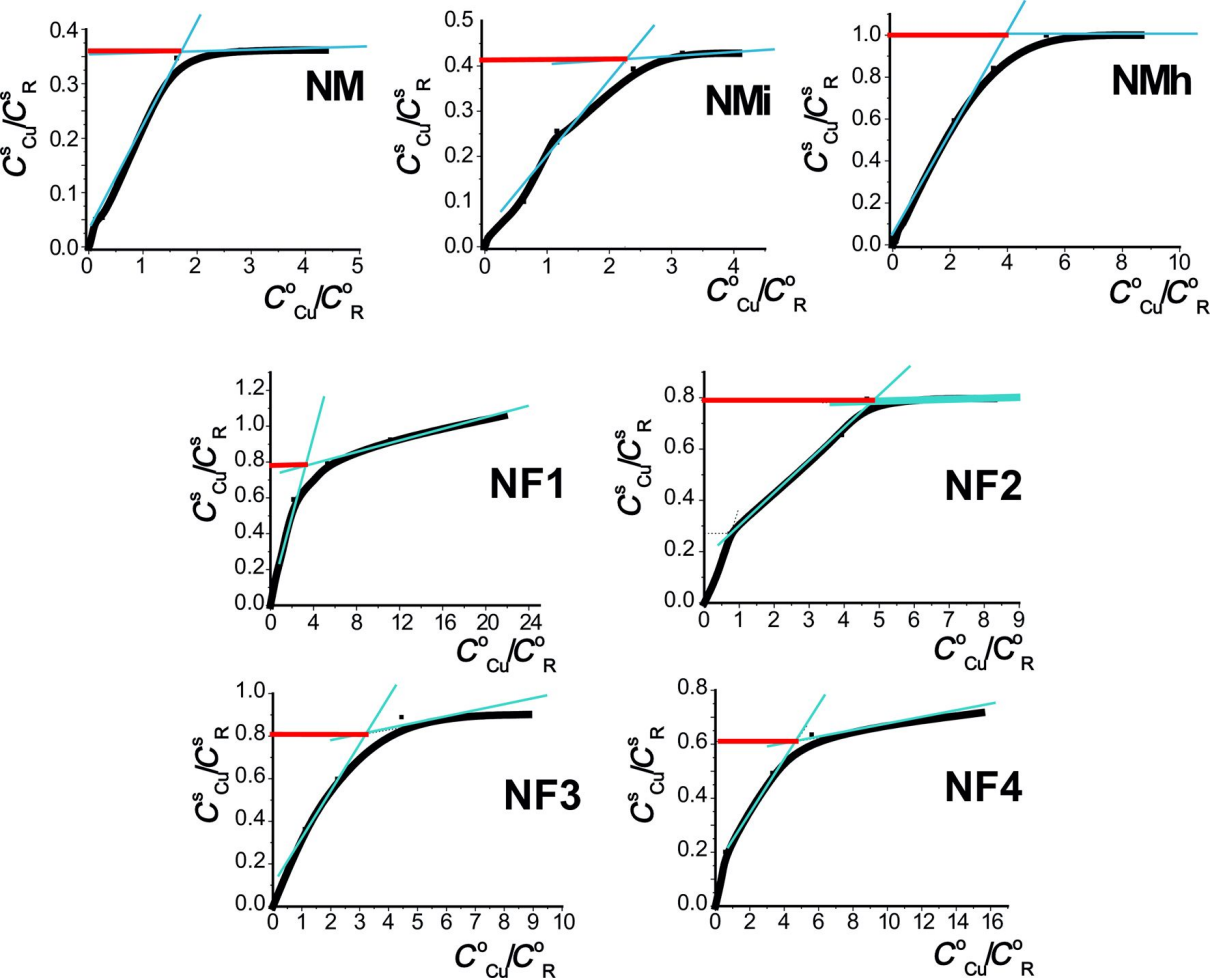
The molar ratio of copper(II)/aminopropyl groups in the surface of the microspheres as a function of that ratio in the initial solution.

To determine the formal coordination surrounding of Cu(II) ions in the adsorption layer, the isotherms were plotted in the form of *C*^s^_Cu_:*C*^s^_R_ as a function of *C*^0^_Cu_:*C*^0^_R_ (Figure 6, Figure S8, Supporting Information File 1).

**Figure 7:**
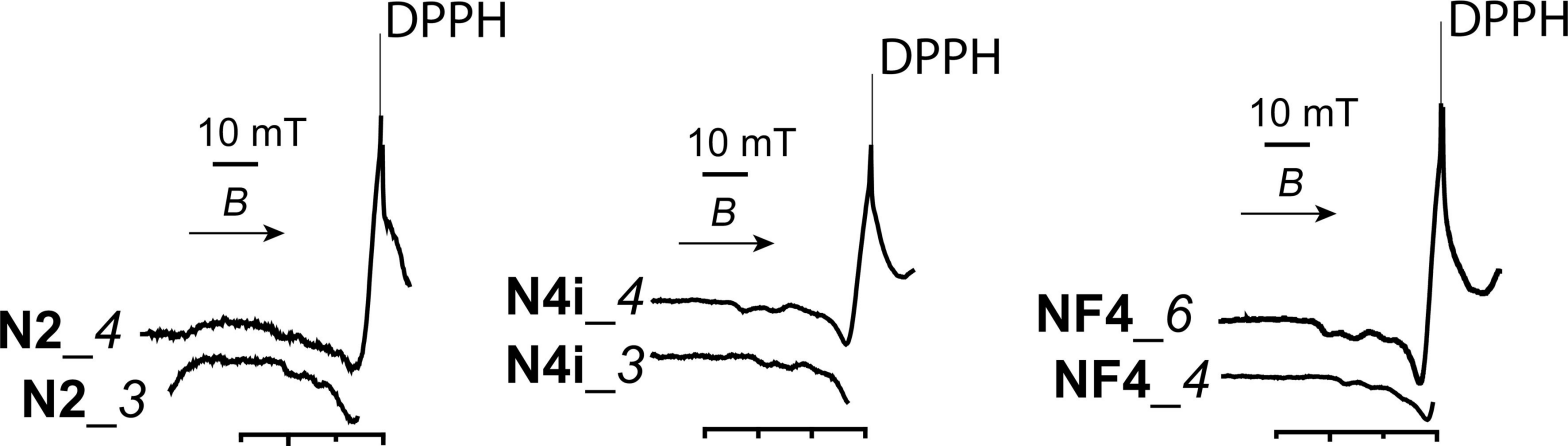
EPR spectra of some samples after adsorption of copper(II) ions (see Table S7, Supporting Information File 1 for details).

**Figure 8:**
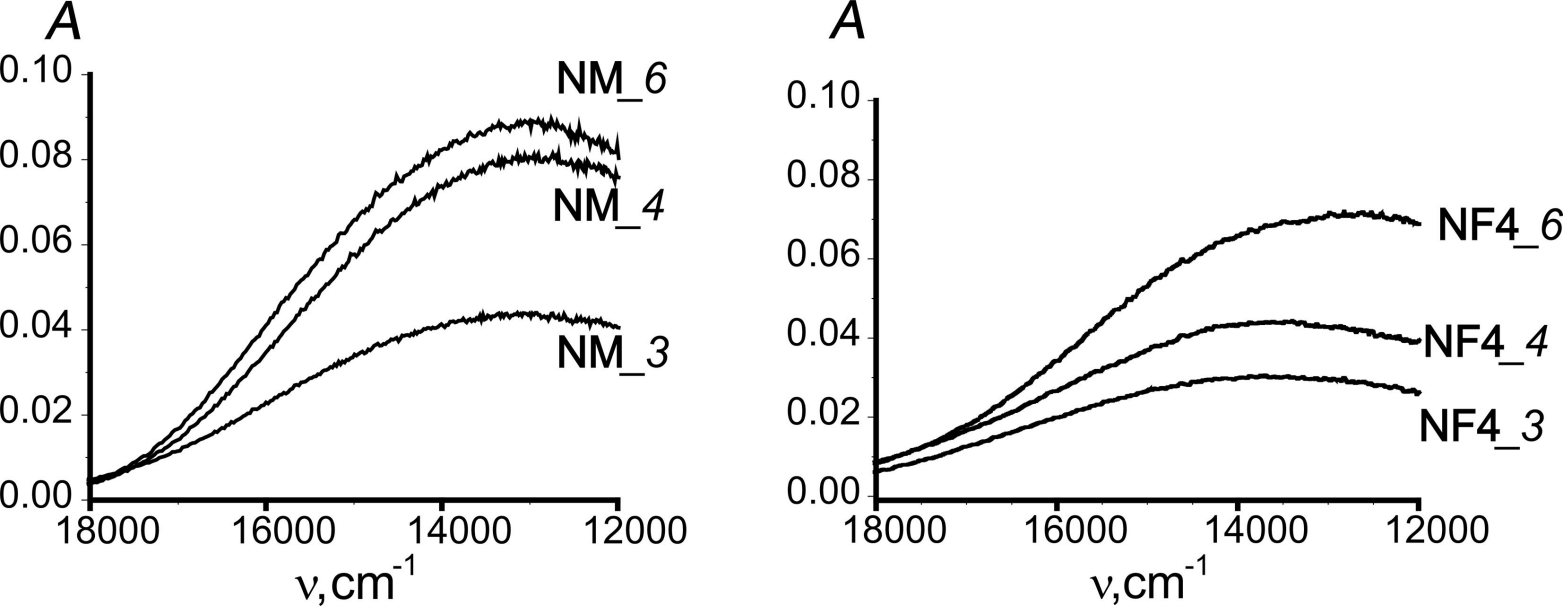
ESDR spectra for samples with different contents of Cu^2+^ in the surface layer.

These data were complemented by EPR and diffuse reflectance spectra (Figure 7 and Figure 8). Generally, the composition of the complexes of copper(II) ions with amine groups of monofunctional samples has a Cu^2+^/Lig ratio in the range of 1:1–1:4 (Table S6, Supporting Information File 1). For bifunctional samples, especially those containing fluoroalkyl radicals, the gross compositions of Cu^2+^/Lig complexes are close to 1:1.

Table S7 (Supporting Information File 1) provides parameters of the EPR spectra of copper(II) complexes formed on the surface of some carriers discussed in this paper, and Figure 7 represents the EPR spectra of some of these systems.

Unfortunately, in some cases, we could not obtain satisfactory EPR spectra, especially when the sorption was carried out from an excess concentration of copper(II) in the solution. But for all the samples, at a molar metal/ligand ratio of 1:2, satisfactory EPR spectra were recorded, the parameters of which (Table S6, Supporting Information File 1) are very close to the values characteristic of the Cu/Lig = 1:2 complex composition. Complexes of such composition also formed at an excess concentration of metal ions (see Table S7, **NF4_***6*, Supporting Information File 1). Similar copper(II) complexes were found on the surfaces of various xerogels containing 3-aminopropyl groups [42].

Figure 8 and Figure S9 (Supporting Information File 1) present ESDR spectra for some samples with different contents of Cu^2+^ ions in their surface layers. All spectra feature a broad band in the region of 13000–20000 cm^−1^, which is characteristic of copper(II) complexes with amino ligands and refers to d→d transitions [43]. The position of the maximum of this band depends on the nature of the functional group and on the *C*^S^_Cu_/*C*^S^_R_ ratio (the degree of surface filling with metal). However, in almost all cases, the maximum of this band is at about 13000–14000 cm^−1^.

We measured adsorption isotherms of *n*-hexane, acetonitrile and water (Figure S10, Supporting Information File 1) to compare the properties of silica nanoparticles with fluorine (see Supporting Information File 1) and amino/perfluoroalkyl-containing bifunctional surface layers. In the case of bifunctional sample **NF2**, at low fillings, the three vapor adsorption isotherms of *n*-hexane, acetonitrile, and water coincide. However, with increasing *P*/*P*_s_ values, motre acetonitrile is adsorbed compared to water and *n*-hexane. During the synthesis, the ratio of amino/fluorine-containing groups was 1:1. Acetonitrile is electrostatically repelled from amino groups of the surface [44]. However, in spite of this, its sorption volume is similar to that of water and *n-*hexane.

## Discussion

Our interest was to identify the molecular mechanisms of how the hydrophobic groups influence stability and reactivity of the aminopropyl groups. We decided to apply additional groups with rather different characteristics to be able to distinguish the principles of their action, namely small alkyl (methyl) groups and rather long perfluoroalkyl ones.

### Factors controlling size and morphology of the particles

Previously it was shown that the introduction of alkyl groups along with 3-aminopropyl groups in the surface layer improves its hydrolytic stability [45] and enhances the adsorption of biomolecules [46–47].

In developing the synthesis in the present work we took into account the already developed principles of the production of APTES-derived materials [48–49]. The particles are generally smaller when higher TEOS/APTES ratios are applied [31]. They are less coalesced in the presence of higher ammonia content because of a stronger negative charging of the growing entities, which is especially important for highly hydrophobic materials that can otherwise form gels separating from solutions [50–52]. The most efficient way to keep the aminopropyl groups on the surface is to add TEOS after the APTES and, especially, to slightly increase the ammonia content [28] (see also Figure S1, Table S1, Supporting Information File 1).

Carrying out the process at higher temperatures resulted in smaller particles that, however, aggregated. MTES is characterized by a more facile hydrolysis and condensation compared to TEOS. This results, in combination with the fact that methyl groups are not charged and in their turn “dilute” the charged groups on the surface, in the observed difference between **N4** and **NM** samples produced under similar conditions. The particle growth facilitated by quick condensation with MTES leads to better shaped particles at lower temperatures, while the increased temperature favors nucleation with less charged and easier coalescing nuclei that aggregate easier (sample **NMh**). The behavior of the three-component system APTES/TEOS/MTES is thus more complex and is not dominated by the reactivity of any single component.

The decrease in TEOS concentration in the reaction solution (at the same PFES/APTES ratio) causes a decrease in particle size (see average diameters of samples **NF3** and **NF1** in Table S1, Supporting Information File 1). Finally, reducing the amount of fluorinated groups compared to **NF3** (Figure 2) leads to the formation of substantially spherical **NF2** (Figure 2). The particles turn more uniform in size with slightly bigger average diameters (Table S1, Supporting Information File 1). The reverse trend is observed in the case of increasing the APTES concentration at a constant TEOS/PFES ratio (see Table S1, samples **NF2** and **NF4**, Supporting Information File 1).

It is important to mention that particles in both **NF4** (Figure 2) and **NM** (Figure 1) samples are considerably smaller in size with of *d*_av_ = 190 nm and 180 nm, respectively, compared to their monofunctional analogues, **N2** (*d*_av_ = 360 nm) and **N4** (*d*_av_ = 280 nm) (Figure S1, Supporting Information File 1). It can be deduced that the introduction of a silane bearing a hydrophobic group possibly favors nucleation over growth and results in relatively smaller particles.

### Molecular structure of the functional layers from IR and NMR data

IR spectroscopy was used to identify the presence of functional groups in the surface layers of nanoparticles. Consequently, IR spectroscopy confirmed the presence of a polysiloxane network containing 3-aminopropyl and methyl or fluorinated functional groups, as well as silanol groups, and alkyl radicals in the synthesized particles. Furthermore, all samples contained water (DRIFT analysis in Supporting Information File 1).

Solid-state CP/MAS NMR spectroscopy, especially ^13^C and ^29^Si NMR spectroscopy, has been widely used to study silica materials, it can provide information about hydrolysis and condensation processes. Clearly, hydrolysis and polycondensation of ethoxysilyl groups significantly accelerated with increasing synthesis temperature [38]. With the introduction of hydrophobic groups, the intensity of the signals from ethoxy groups (at 58–60 ppm and a shoulder at 18–19 ppm) in the ^13^C CP/MAS NMR spectra (Figure 3a, Table S2, Supporting Information File 1) is significantly reduced. As for the characteristic signals from ethoxysilyl groups in the spectrum of fluorinated sample, they are faint, indicating completeness of their hydrolysis during the synthesis. Apparently, the ethoxy groups constitute a part of the interface between the particles and the solution. Their weak hydrophobic interactions with the ethanol-based solvent are apparently outcompeted by either stronger hydrogen bonding to the amino/ammonium functions or the strong hydrophobic interactions involving the fluoroalkyl groups.

Thus, taking into account the ^13^C CP/MAS NMR data, it may be concluded that R = Н for the fluorinated samples, while for other samples R = ethyl radical in structural units (Si(O_0.5_)_2_(ОR)_2_ or (Si(O_0.5_)_3_(ОR). According to ^29^Si CP/MAS NMR data the cores of the synthesized particles are likely to consist of condensed tetrahedral SiO_4_, and their surface layer contains functional and silanol groups belonging to structural units of different composition.

### Probing the ligand layer structure by Cu(II) adsorption

There are several key factors affecting the adsorption of ions on the surface, namely the content, availability, and arrangement of functional complexing groups. The content of functional groups was determined by various physical methods (Table S4, Supporting Information File 1). For all the samples, the content of amino groups calculated from elemental analysis is higher than determined by titration, which means that not all amino groups are available for the sorption of ions. It may be connected with the partial aggregation of particles. The arrangement of amino groups in the surface layer of bifunctional silica particles depends on the length and nature of the second functional group.

The topography of the functional groups attached to the surface of the amorphous carriers, and their conformational behavior are the least extensively studied matters so far. This prevents the fully reliable interpretation of the experimental results and hinders the prediction of the behavior of such functionalized carriers in various chemical processes. Such limited knowledge results from the intricacy of the problem, which requires the application of different physical methods for its solution. We used the highly reliable metal-probing method. Copper(II) ions were chosen as metal ions for probing, since the composition and structure of copper–ammonia complexes are well known. They also correlate well with the data obtained by some other physical methods (e.g., electron spectroscopy and EPR) as is traced in detail in [53].

**Figure 9:**
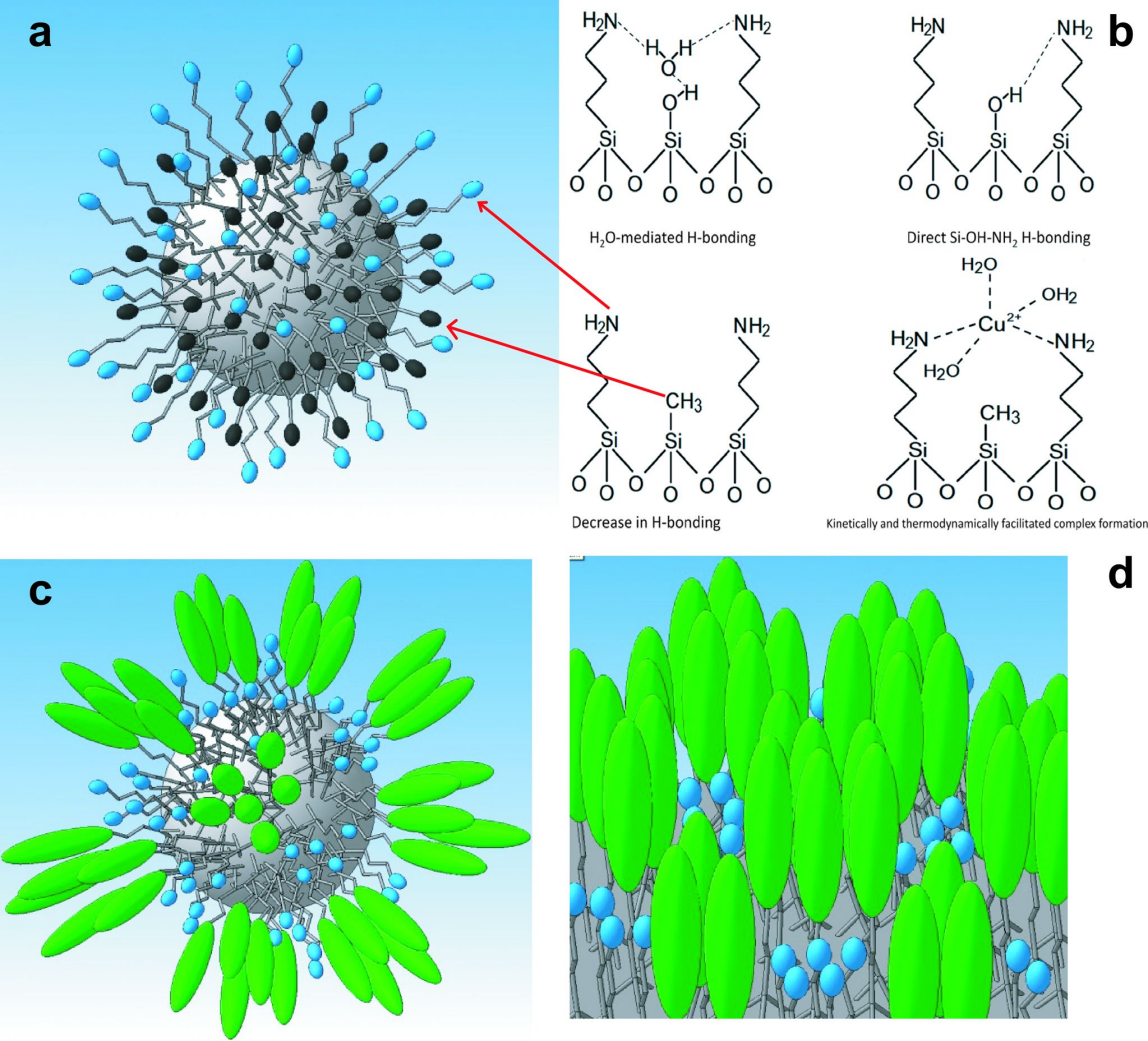
а) Proposed structure of the surface layer on the of bifunctional silica particles bearing methyl and aminopropyl groups (in the image chains with black and blue headgroups, respectively); b) scheme of molecular interactions in the monofunctional layer bearing aminopropyl groups and the bifunctional layer with aminopropyl and methyl groups; c,d) proposed surface structure of the bifunctional layer for particles bearing perfluoroctyl and aminopropyl (in the image chains with green and blue head groups, respectively) groups on the surface.

Comparing the values of the sorption rate constants for monofunctional and amino/methyl samples, we can conclude, that the rate of sorption for the latter is almost twice as high (Figure 4, Table S5, Supporting Information File 1). This may be due to the fact that amino groups located on the surface of monofunctional amino silica particles can form hydrogen bonds with silanol groups. In contrast, the amino groups on the surface of amino/methyl silica particles are surrounded by methyl groups, which prevent the formation of hydrogen bonds with silanol groups (Figure 9a,b), increasing the speed of interaction with copper(II) ions. As for amino/fluorine-containing particles, their smaller rate constant values (Table S5, Supporting Information File 1) indirectly indicate the “island-like” structure of the surface layer, where the “islands” of amino groups are surrounded with long hydrophobic radicals (Figure 9c,d) preventing the diffusion of ions to the centers of adsorption. Consequently, the introduction of methyl groups thus either stabilizes the surface layer or leads to enhanced accessibility of amino groups. The latter correlates well with the data from the adsorption of Cu^2+^ cations (see below).

The composition of complexes depends on several factors. For example, the composition of complexes for the samples with amino/methyl groups obtained at different temperatures appeared to be different. Thus, for the sample **NMi**, the Cu^2+^/Lig ratio is close to 1:2, which is similar to xerogels synthesized on an ice bath [54], where amino groups are located on the surface in pairs. When the synthesis temperature is higher (sample **NMh**), the rate of condensation is also higher, and more methyl groups get fixed on the surface according to TGA and elemental analysis data (Table S1 and Table S4, Supporting Information File 1). Therefore, some of the amino groups are located at a distance that does not favor the formation of stable 1:2 complexes, and the form dominating at higher Cu^2+^/Lig ratios can be assumed to have the 1:1 composition. As for the sample **NM**, synthesized at room temperature, it appears that the ratio of copper(II) ions to aminopropyl groups in the upper layer is 1:3. It may be explained by the presence of the combination of Cu^2+^/Lig complexes 1:2 and 1:4 (Figure 6).

This is consistent with the 1:2 composition of Cu^2+^/Lig complexes suggested by the EPR spectra. According to literature [43,55], the tetragonally distorted octahedral copper(II) complexes, which contain four amine ligands in the equatorial plane are characterized by a band of d→d transitions around 17000–19000 cm^−1^, whereas ESDR spectra of the complexes with two amine ligands in the equatorial plane exhibit a band in the region of 14500–15000 cm^−1^. This suggests that the coordination sphere of copper(II) ions in the surface layers of the discussed samples contains two amino groups. The shift of the maximum to lower frequencies may result from the distortion of the octahedral coordination of the central atom. Obviously, with increasing *C*^S^_Cu_/*C*^S^_R_ ratio the sorption will also take place in the surface regions less favorable for the formation of ideal coordination polyhedra. The calculations of the Langmuir and Freundlich isotherm sorption parameters are presented in Table S6 (Supporting Information File 1). According to Table S6, the adsorption isotherms for amino and amino/methyl samples fit well the Freundlich isotherm model, whereas the fluorine-containing samples fit the Langmuir isotherm model.

### Correlation between the structure of the surface layers and adsorption of small molecule substrates

As it can be deduced from the results of EPR and ESDR studies, the surface of the particles is energetically and chemically heterogeneous due to the presence of different functional groups, arranged either as a “random forest” in the samples bearing methyl group, or as islands with distinctly different properties and structures. Therefore, such a surface is predisposed for specific adsorption interactions with molecules of different electronic structure and especially different polarity. The molecules of *n-*hexane are capable of adsorption interactions due to dispersion forces, whereas interaction of acetonitrile with the surface is dominated by the basic properties of the nitrogen atom.

Obviously, acetonitrile (as well as *n-*hexane) interacts with hydrophobic sites formed by fluorine-containing groups, while the water molecules interact with hydrophilic sections created by amino groups. Thus the introduction of aminopropyl groups in the fluorine-containing surface layer changes the nature of the surface, which is confirmed by the order of the adsorption isotherms in Figure S10 (Supporting Information File 1). But because of the long length of the fluorinated “tails”, 3-aminopropyl groups do not overhang on the surface, which promotes the adsorption of acetonitrile by such bifunctional samples.

Thus, we can assume that the affinity to acetonitrile is probably related to its sorption on hydrophobic “islands” formed by fluorine-containing “tails”. Furthermore, the values of sorption volume of the vapors of *n*-hexane, acetonitrile, and water for sample **NF2** are similar, which indirectly confirms the “island” structure of the surface layer (Figure 9c,d).

## Conclusion

Silica particles with bifunctional surface layers and high content of available aminopropyl groups were successfully produced by a modified one-step Stöber approach. Combining hydrophobic ligands with the aminopropyl grous in the surface layers of hybrid silica particles appears to offer generally smaller particles. Their surface is apparently porous when methyl groups are introduced as co-ligand and becomes smoother when perfluoroalkyl groups are applied, especially when their coverage fraction is increased. The molecular structure of the bifunctional layers depends strongly on the nature and fraction of the co-ligand. The methyl groups dilute the aminopropyl groups uniformly, opening for uptake of higher amounts of Cu^2+^ ions used as probes in relation to the amount of grafted amino functions. This should even improve potentially the reactivity used for further functionalization of amino modified silica. The introduction of perfluoroalkyl co-functions even at low fraction leads to the formation of “island” structures. Increase in the content of the perfluoroalkyl co-ligands leads to huddling and inactivation of amino functions. As a consequence the amino functions become apparently partly inaccessible for the complexation with Cu^2+^ as testified by low Cu^2+^/Lig ratios (0.35 and lower), while the simultaneous EPR and ESDR measurements reveal the formation of the trans Cu(Lig)_2_^2+^ complexes. Changes in the surface geometry in the bifunctional layers even result in unusual patterns of adsorption of small organic molecules, making the particles capable to use both hydrophobic and hydrophilic functions simultaneously.

## Experimental

### Materials

The applied precursors were: tetraethoxysilane, Si(OC_2_H_5_)_4_ (TEOS, 98%, Aldrich); 3-aminopropyltriethoxysilane, (C_2_H_5_O)_3_Si(CH_2_)_3_NH_2_ (APTES, 98%, Fluka); methyltriethoxysilane, (C_2_H_5_O)_3_SiCH_3_ (MTES, 99%, Aldrich); 1*H*,1*H*,2*H*,2*H*-perfluorooctyltriethoxysilane, (C_2_H_5_O)_3_Si(CH_2_)_2_(CF_2_)_5_CF_3_ (PFES, 98%, ABCR); ethanol (96%); aqueous ammonia (25% aq) (reagent grade, Macrochem, Ukraine). Reagents for acid–base titration and sorption: Cu(NO_3_)_2_·3Н_2_О (reagent grade, Macrochem, Ukraine), NH_4_Cl, NaNO_3_, NaCl (chemically pure, Macrochem, Ukraine); HNO_3_, HCl, NaOH, EDTA - fixanal concentrates (Reahim, Ukraine); murexide (analytical grade, Reahim, Ukraine).

### Synthesis of nanoparticles

The particles were produced by a single-step Stöber approach (for details see Supporting Information File 1) using TEOS, APTES and an additional hydrophobic reagent and differed either in the ratio of the reactants or in the temperature conditions.

Methyl-substituted samples were synthesized using a ratio of TEOS/APTES/MTES = 3:0.5:0.5 at different temperatures and are denoted as **NM** (RT.), **NMi** (0 °C, ice bath), and **NMh** (50 °C). Perfluorooctyl-substituted samples were produced at room temperature at different reactant ratios and are denoted as **NF1**, **NF2**, **NF3**, and **NF4** (TEOS/APTES/PFES = 3:0.25:0.25, 3:0.5:0.1; 3:0.5:0.5, and 3:1:0.1, respectively).

For the sake of comparison monofunctional samples were produced, denoted as **N1** (TEOS/АРТЕS = 1:1, RT), **N2***, ***N3** and **N4** (TEOS/APTES = 3:1, RT; the first with addition of first APTES, the second with first addition of TEOS, and the third with later addition of the NH_3_ catalyst). The labels **N4i** and **N4h** stand for carrying out the **N4** procedure at 0 °C and 50 °C, respectively. The labels **F1** and **F2** stand for the RT procedures with TEOS/PFES = 3:1 and 3:0.5, respectively.

### Characterization techniques

Thermal analysis was performed on the MOM Q-1500 D (Paulik–Paulik–Erdey) derivatograph operating in the range of 20–1000 °C, with a heating rate of 10 °C·min^−1^.

DRIFT spectra were recorded on a Thermo Nicolet Nexus Fourier-transform infrared spectrometer in the range of 400–4000 cm^−1^, working in "Nexus Smart Collector" mode and averaging 50 scans with a resolution of 8 cm^−1^. The samples were previously ground with solid KBr (Spectral, Aldrich).

^13^C and ^29^Si MAS NMR experiments were carried out on a Bruker Avance II 400 spectrometer using 4 mm rotors (ZrO_2_) spun at 10 kHz. ^13^C NMR spectra were recorded using ^1^H→^13^C CP/MAS, ^1^H decoupling during acquisition, 3 ms contact time, and 5 s recycling delay. The number of scans was between 768 and 2300. ^29^Si NMR spectra were recorded with 3 µs excitation pulses, a contact time of 2 ms, and 5 s recycling delay. The number of scans was between 640 and 1024. ^13^C and ^29^Si chemical shifts are referenced towards 4,4-dimethyl-4-silapentane-1-sulfonic acid (DSS).

CHNS elemental analysis was performed by elementary analyzer Vario MACRO cube (Elementar Analysensysteme GmbH, Germany) using a thermal conductivity detector. Helium and oxygen (both purity 99.995%) were used as the carrier and combusting gases, respectively, with 2 bar intake pressure. The combustion tube was set at 1150 °C and the reduction tube at 850 °C. Sulfanilamide C_6_H_8_N_2_O_2_S was used as CHNS standard.

For SEM studies with a JSM-6060LA analytical scanning electron microscope (Jeol, Tokyo, Japan) using secondary electrons at an accelerating voltage of 30 kV, the samples were fixed on the objective tables. To prevent the accumulation of the positive charges and to receive contrasting images, the surface of the samples was covered with a thin continuous layer of gold or platinum by cathodic sputtering in vacuum. The morphology of the obtained samples was also studied by Hitachi TM-1000 tabletop microscope capable of energy-dispersive X-ray spectroscopy (EDXS) analysis.

The measurement of nitrogen adsorption isotherms was carried out on a Kelvin-1042 (Costech Microanalytical) analyzer. The time for preliminary degassing was 1 h at 110 °C. The BET surface area [55] was evaluated at relative pressures of 0.05–0.35.

Adsorption isotherms of *n*-hexane, water and acetonitrile were obtained at 20 °C using a vacuum microbalance (balance sensitiveness: 1.9–2.8 mg/mm). The samples were first evacuated at 105 °C to a constant weight. Air was removed from the adsorbate by cycles of freezing/defreezing during vacuum pumping.

Acid–base titration was used to determine the content of amino groups [56]. This method is based on the determination of the number of protons captured by amine groups after submerging the sample batch (0.1 g) in excess of 0.01–0.05 M HCl till equilibrium was established (which was 24 h). The excess of HCl was determined by back titration with 0.01–0.05 M NaOH solution (using methyl orange as indicator).

The study of the equilibrium time for copper(II) sorption was performed using 0.02 g of sorbent at 28 °C. 20 сm^3^ of solution containing double excess of copper(II) in relation to stoichiometry was added, varying only the contact time from 5 min to 24 h. The linear pseudo-first-order and pseudo-second-order equations [57] were used for the description of the kinetic model:


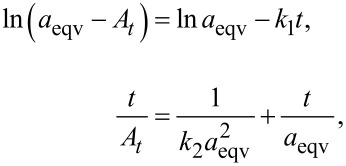


where *A_t_* and *a*_eqv_, are the adsorbed amounts at time *t* and at equilibrium (mmol/g), respectively; *k*_1_ and *k*_2_ are the rate constants of pseudo-first-order (min^−1^) and pseudo-second-order adsorption process (g·mmol^−1^·min^−1^).

The sorption of copper(ІІ) ions from water solution was studied under static conditions at 28 °C. The sorbent batch of 0.03 g was placed in a 50 сm^3^ weighing bottle, and 20 сm^3^ of Cu(NO_3_)_2_ solution was added to it. The ionic strength was maintained by 1 M NaNO_3_ solution. The concentration of metal ions in aqueous medium was determined by direct titration of metal ions with 0.0125–0.025 M EDTA (indicator: murexide, buffer: ammonia).

Isotherm model evaluation: the Langmuir isotherm is a broadly used model, assuming adsorption to occur on specific sites uniformly spread on the surface of the adsorbent. It is commonly used for description of processes, where adsorbed species form a monolayer, and is described by the following equation:


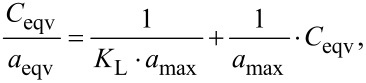


where *C*_eqv_ is the concentration of solute remaining in solution after equilibrium to be reached (mmol/L); *a*_eqv_ is the amount of solute adsorbed under the same conditions (mmol/g); *a*_max_ is the maximum adsorption capacity in the monolayer and *K*_L_ is the equilibrium constant of the adsorption process.

The Freundlich isotherm describes multilayer adsorption and has been used to establish a mathematical relationship between the amounts of solute adsorbed and its concentrations in solution at equilibrium:


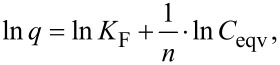


where *q* is the amount of solute adsorbed (mmol/g); *C*_eqv_ is the concentration of solute remaining in solution after equilibrium (mmol/L); *K*_F_ and 1/*n* are parameters related to maximum adsorption capacity in the multilayer of the adsorbent [58–59].

X-band EPR spectra of the samples were recorded at room temperature using a radio spectrometer РE-1306 equipped with a frequency meter ChZ-54 and frequency converter YaZCh-87. The magnetic field was calibrated using 2,2-diphenyl-1-picrylhydrazyl (DPPH) (*g* = 2.0036) and ions of Mn^2+^ in MgO matrix (*g* = 2.0015). The sorption of copper(II) ions on the samples analyzed with EPR was conducted from acetonitrile solutions.

Electron spectra of diffuse reflectance (ESDR) of the amino-functionalized particles containing copper(II) ions were recorded on a spectrophotometer Specord UV–vis (model M-40).

## Supporting Information

File 1 Additional experimental data.
