# Supplementary material for: Tailoring bifunctional hybrid organic–inorganic nanoadsorbents by the choice of functional layer composition probed by adsorption of Cu2+ ions
Source: Beilstein J Nanotechnol. 2017 Feb 2;8:334–47. doi: 10.3762/bjnano.8.36 (PMC5301806; doi:10.3762/bjnano.8.36)
Supplement: File 1 — Additional experimental data. [file Beilstein_J_Nanotechnol-08-334-s001.pdf]

Supporting information

for

Tailoring bifunctional hybrid organic–inorganic nanoadsorbents by the choice of functional layer composition probed by adsorption of Cu^{2+} ions

Veronika V. Tomina[1], Inna V. Melnyk[1,2], Yuriy L. Zub[1], Aivaras Kareiva[3], Miroslava Vaclavikova[2], Gulaim A. Seisenbaeva*[4] and Vadim G. Kessler[4]

Address: [1]Chuiko Institute of Surface Chemistry of NASU, 17, Generala Naumova Str., Kyiv 03164, Ukraine, [2]Institute of Geotechnics SAS, 45, Watsonova, Kosice 04001, Slovak Republic, [3]Department of Inorganic Chemistry, Vilnius University, 24, Naugarduko Str., Vilnius LT-03225, Lithuania, and [4]Department of Chemistry and Biotechnology, Swedish University of Agricultural Sciences, 8, Almas allé, Uppsala 75007, Sweden

Email: Gulaim A. Seisenbaeva* - gulaim.seisenbaeva@slu.se

* Corresponding author

Additional experimental data

Synthesis of nanoparticles

Synthesis of monofunctional nanoparticles with amine-containing groups in the surface layers

Sample ***N1*** (TEOS/APTES = 1/1 (mol.)). 21.7 ml of 25% aq. NH_4OH and 14 ml of distilled water were added at constant stirring to 100 ml of ethanol. In several minutes, 6 ml (0.026 mol) APTES were added to the mixture. The solution immediately turned cloudy; however, in few minutes it got transparent again. Then 6 ml (0.027 mol) of TEOS were added to it. In two minutes, the solution was cloudy again, and the amount of precipitate started increasing. The suspensions was stirred for 1 h, and the precipitate was centrifuged (for 10 min at 5000 rpm), and washed triply with ethanol. The sample was dried in the drying oven at 100°C for 3 days. The yield was 2.3 g.

Samples ***N2*** *and* ***N3*** (TEOS/APTES = 3/1). The synthesis was carried out similar to **N1** (APTES volume was 2 ml (0.0085 mol)), but in the case of ***N3*** TEOS was added firstly to the mixture of EtOH, NH_4OH and H_2O and in one minute APTES was added. The yields of the resulting white powders were 1.87 g and 1.79 for ***N2*** and ***N3*** respectively.

Sample ***N4*** (TEOS/APTES = 3/1). 4 ml of TEOS and 1.4 ml of APTES were added to 100 ml of ethanol at room temperature (22°C). After the appearance of opalescence in 30 min, there were added 1.9 ml of 25% aq. NH_3. Opalescence was increasing over time. In 1 h, white precipitate was centrifuged and washed as in previous syntheses. The yield was 0.56 g. Anal clcd for $(H_2N(CH_2)_3SiO_{3/2})(SiO_2)_3$: N, 4.67. Found: N 4.08.

Sample ***N4i*** (TEOS/APTES = 3/1). The synthesis was conducted similar to ***N4*** synthesis, but in an ice bath at a temperature of 3-4°C. Opalescence

began in 100 min, after which ammonia was added. In 3 h after ammonia addition, the precipitate was separated by centrifugation and washed. Last 2 h it was gradually heating to room temperature. The yield was 0.82 g. Anal clcd for $(H_2N(CH_2)_3SiO_{3/2})(SiO_2)_3$: N, 4.67. Found: N 3.02.

Sample ***N4h*** (TEOS/APTES = 3/1). The synthesis was conducted similar to ***N4***, but with heating to 50°C. Opalescence began in 15 min, after which ammonia was added. In 1 h after ammonia addition, the precipitate was centrifuged and washed. The yield was 0.82 g. Anal clcd for $(H_2N(CH_2)_3SiO_{3/2})(SiO_2)_3$: N, 4.67. Found: N 4.62.

Synthesis of monofunctional nanoparticles with fluorine-containing groups in the surface layer

Sample ***F1*** (TEOS/PFES = 3/1). 2.67 ml (0.012 mol) of TEOS and 1.53 ml (0.004 mol) of PFES were dissolved in 2.33 ml of ethanol. The solution of ammonium hydroxide in ethanol (3.71 ml of 25% aq. NH_4OH in 31.84 ml ethanol) was added drop-wise and under constant stirring to the mixture, and left stirring for 2.5 h. In the beginning, the solution was transparent, but later the formation of particles was observed. Precipitate was separated by centrifugation (for 10 min at 6000 rpm), washed with ethanol and again centrifuged. The washing procedure was repeated twice. The sample was dried in oven at 100°C to constant mass. The sample featured white powdery substance. The yield was 2.08 g. Anal clcd for $CF_3(CF_2)_5(CH_2)_2SiO_{3/2})(SiO_2)_3$: C, 16.6; H, 0.7. Found: C, 16.5; H, 1.2.

Sample ***F2*** (TEOS/PFES = 3/0.5) was synthesized similar to ***F1*** (with PFES amount of 0.77 ml (0.002 mol)). It featured white powdery substance. The

yield was 1.22 g. Anal clcd for $CF_3(CF_2)_5(CH_2)_2SiO_{3/2})(SiO_2)_6$: C, 12.6; H, 0.5. Found: C, 12.6; H, 1.6.

Synthesis of bifunctional nanoparticles with hydrophobic (perfluorooctyl-, methyl-, or n-propyl-) and amine-containing groups in the surface layer

Sample ***NM*** (TEOS/APTES/MTES = 3/0.5/0.5). Its synthesis was carried out similar to ***N4****,* but APTES and MTES volumes were 0.7 ml and 0.6 ml. After the appearance of opalescence in 40 min, ammonia was added. In 3 h after addition the precipitate was centrifuged and washed. The yield was 0.89 g.

Sample ***NMi*** (TEOS/APTES/MTES = 3/0.5/0.5). Its synthesis was carried out similar to ***NM***, but in an ice bath at a temperature of 3-4°C. After the appearance of opalescence in 60 min, ammonia was added. In 3 h after addition the precipitate was centrifuged and washed. The yield was 0.52 g.

Sample ***NMh*** (TEOS/APTES/MTES = 3/0.5/0.5). Its synthesis was carried out similar to ***NM***, but with heating to 50°C. After the appearance of opalescence in 15 min, ammonia was added. In 1 h after addition the precipitate was centrifuged and washed. The yield was 0.49 g.

Sample **NF1** (molar ratio TEOS/APTES/PFES = 3/0.25/0.25). 0.234 ml (0.001 mol) of APTES were added to the solution of ammonium hydroxide in ethanol (3.71 ml of 25% aq. NH_4OH in 31.84 ml of ethanol). After the disappearance of turbidness, the solution was added, under constant stirring, to the mixture of 2.67 ml (0.012 mol) of TEOS, 0.384 ml (0.001 mol) of PFES, and 2.33 ml of ethanol, and left stirring for 1.5 h. In the beginning, the solution was transparent, but in 10 min the formation of particles was observed. Precipitate was centrifuged (for 10 min at 6000 rpm), washed with ethanol and

again centrifuged. The washing procedure was repeated twice. The sample was dried in oven at 100°C to constant mass. The sample featured white powdery substance. The yield was 0.85 g.

*Samples **NF2, NF3** and **NF4*** (molar ratio TEOS/APTES/PFES/= 3/0.5/0.1; 3/0.5/0.5 and 3/1/0.1, respectively) were synthesized similar to ***NF1*** (with PFES:APTES ratio of 0.153 ml (0.0004 mol):0.47 ml (0.002 mol); of 0.77 ml (0.002 mol):0.47 ml (0.002 mol) and of 0.153 ml (0.0004 mol):0.94 ml (0.004 mol), respectively). The yields of the resulting white powders were 0.8 g, 1.39g and 1.1g for ***NF2, NF3*** and ***NF4***, respectively.

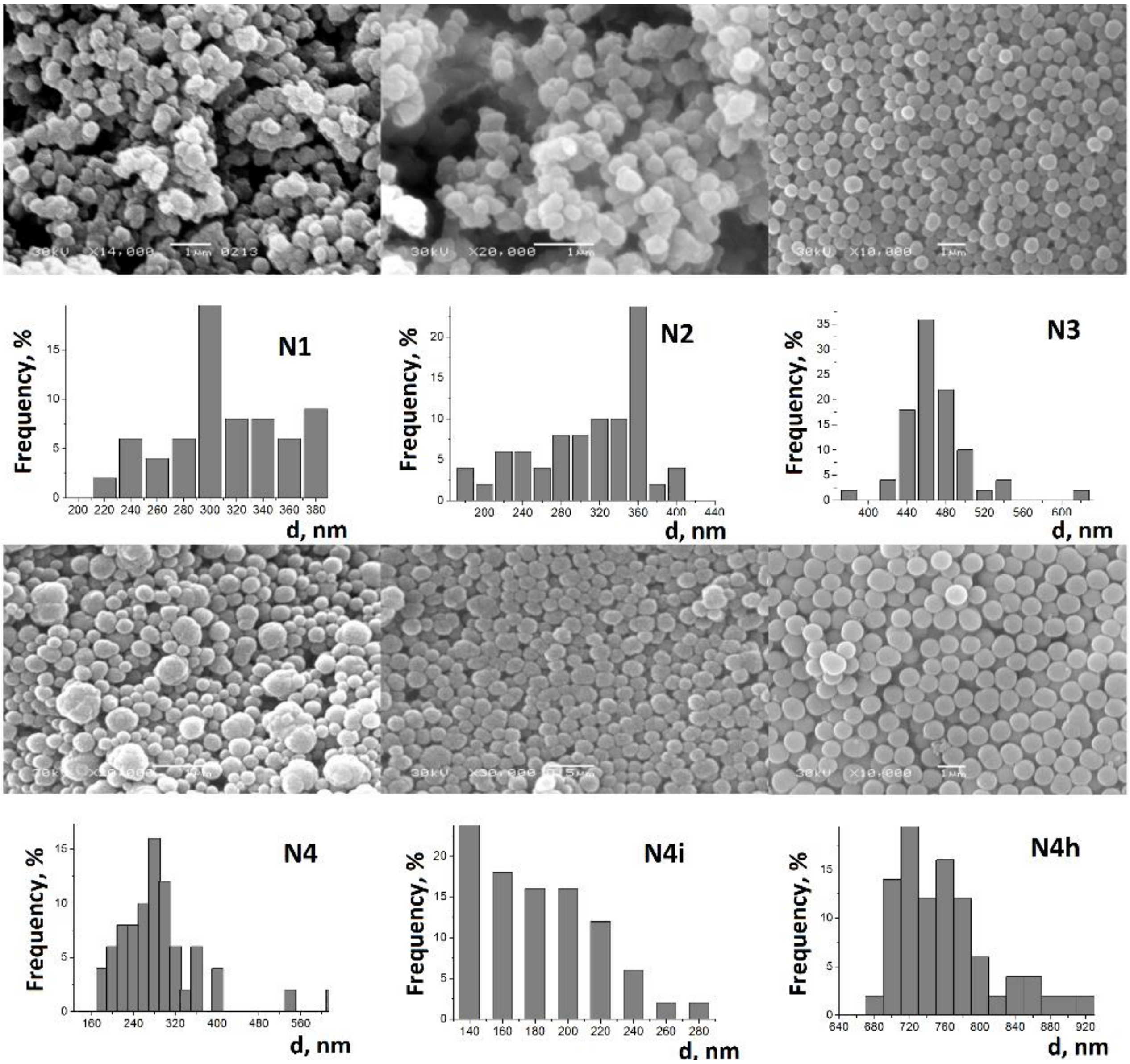

Figure S1: SEM images and particle size distribution curves for monofunctional amino-containing samples ***N1***, ***N2***, ***N3***, ***N4***, ***N4i***, ***N4h***.

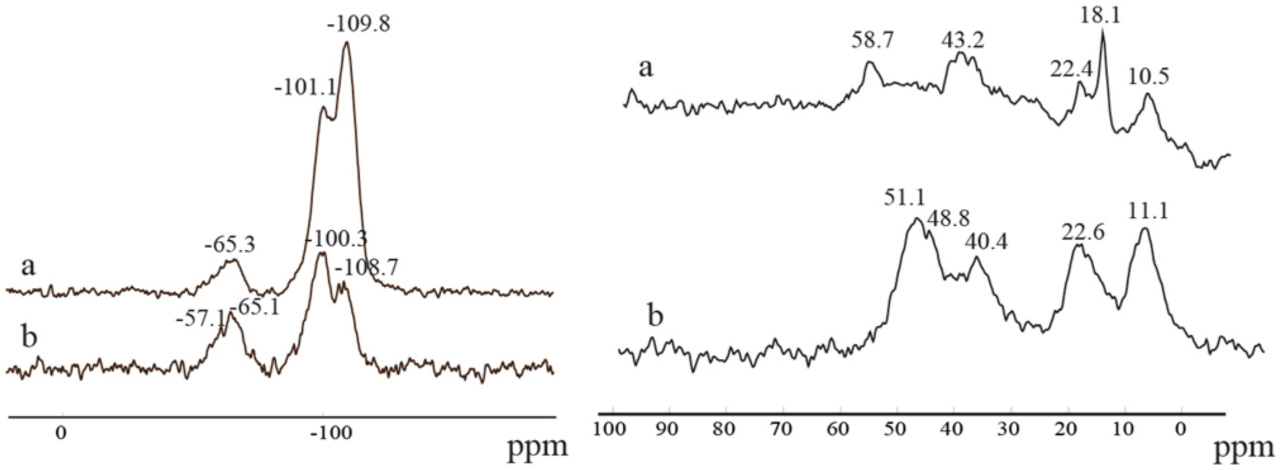

Figure S2: ^{29}Si CP/MAS (left) and ^{13}C CP/MAS NMR spectra of compounds with amine groups: a – ***N2***, b – ***N5****.*

Characterization of fluoroalkyl derived monofunctional samples

SEM microphotographs of synthesized samples with fluorine-containing groups also confirm the formation of nanoparticles (see Fig. FS3). Considering particles with monofunctional fluorine-containing surface layer (samples ***F1*** and ***F2***), the increase in the content of PFES in the reaction mixture produced more uniform particles (***F1***, Fig. FS3) bigger in size (Table TS1). The surface of sample ***F2*** is rough and each particle seems to be composed of smaller particles (10-20 nm in size) (Fig. FS3). Meanwhile sample ***F1*** with higher relative content of PFES has smooth surface and features particles close to spherical (Fig. FS3). Moreover, relative content of fluorine determined by EDXS analysis for both samples ***F1*** and ***F2*** correspond to the initially desired F/Si relations (Fig. FS4). As well as the content of perfluoroctyl groups, recalculated from elemental analysis on carbon for samples ***F1*** (1.7 mmol/g) and ***F2*** (1.3 mmol/g) also coincide with the theoretically assessed values based on the ratios of reacting alkoxysilanes (1.73 mmol/g for ***F1*** and 1.32 mmol/g for ***F2***). It should be

mentioned that specific surface of sample ***F1*** is significantly less than the sample ***F2*** (see Table TS1). Consequently, higher density of surface groups on the surface of sample ***F1*** promotes the formation of smoother spherical particles, apparently via the hydrophobic interactions. Due to the above-mentioned different particle structures of samples ***F1*** and ***F2*, ** their size comparison would be incorrect.

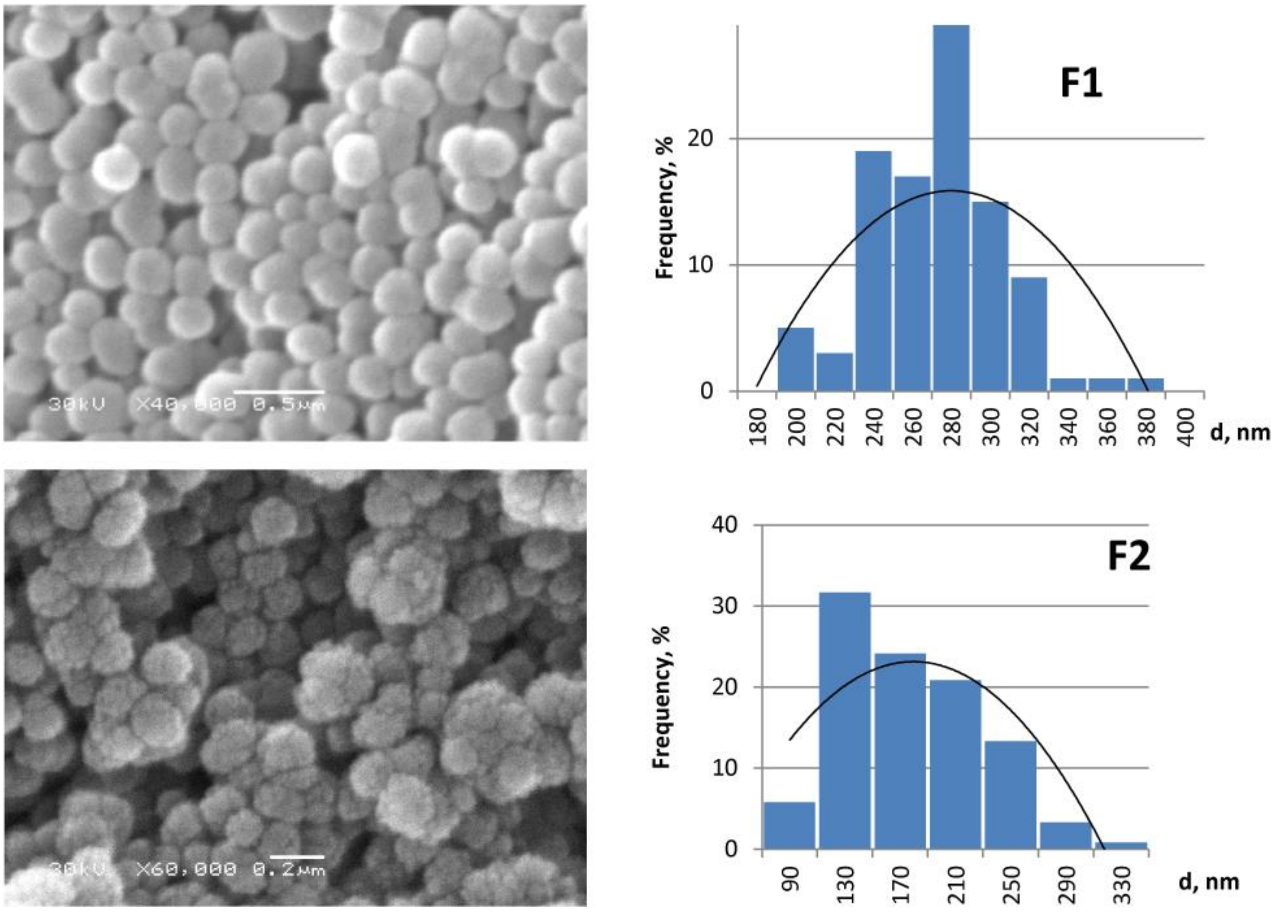

Figure S3: SEM images of fluoroalkyl substituted samples.

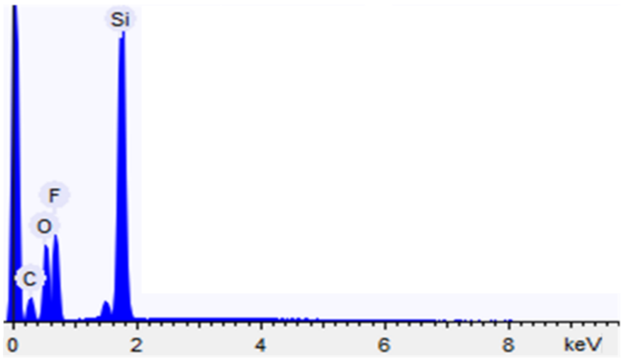

Figure S4: The EDXS analysis of sample ***F1***.

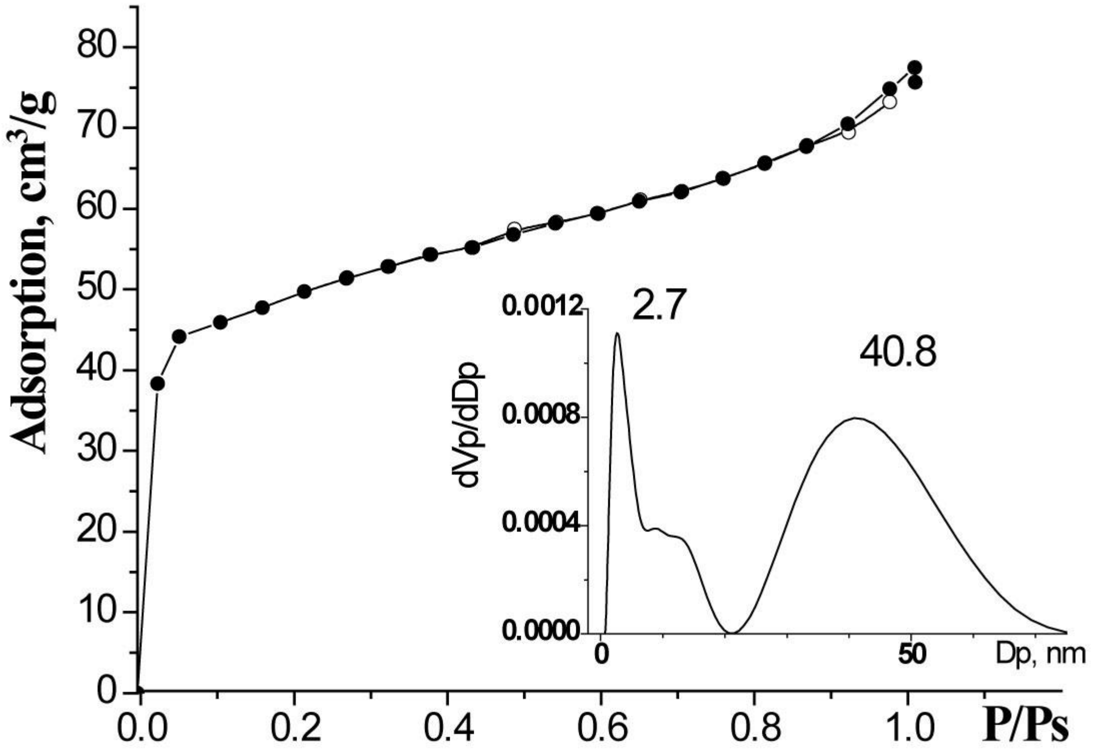

Figure S5: Nitrogen adsorption-desorption isotherm and pore-size distribution curve for sample ***F2***.

DRIFT analysis of the surface layers

The assignment of absorption bands in the DRIFT spectra of samples was carried out using references [1-3]. In the DRIFT spectrum of the sample ***N2*** (Fig. FS6, spectrum 1) the absorption band at 1534 cm^{-1}, resulting from $\delta(NH_2)$ bending of the amino groups is clearly visible. In addition, the DRIFT spectrum also contains an intense absorption band with a high-frequency shoulder in the region of 1000-1200 cm^{-1}, which is characteristic of the v_{as}(SiOSi) stretching vibrations. This indicates the formation of a network of polysiloxane bonds. The band of medium intensity at 1638 cm^{-1} refers to $\delta(H_2O)$ bending. The presence of propyl chains ($Si–CH_2CH_2CH_2–N$) in the DRIFT spectra is indicated by a group of adsorption bands of weak intensity in the region 1390-1440 cm^{-1} and two adsorption bands of medium intensity in the region 2800-3000 cm^{-1}. They are typical of CH_2 bending and of stretching vibrations of CH, respectively. Note the presence of the low-intensity absorption band at 1412 cm^{-1} (see Fig. FS6), which refers to $\delta(Si–CH_2)$ vibrations of 3-aminopropyl moiety. The DRIFT spectra of the other samples with amino groups are identical to the described above.

Interestingly, heating the ***N2*** sample in vacuum to a temperature of 100°C leads to the disappearance in the DRIFT spectrum of the absorption bands at 1638 cm^{-1} (Fig. FS6, spectrum 2) and 1534 cm^{-1}, and the appearance of the absorption band at 1580 cm^{-1}. The shift of the absorption band from 1534 cm^{-1} to 1580 cm^{-1} indicates different surrounding of the amino groups at different temperatures. Thus, the absorption band at 1534 cm^{-1} is characteristic of amino groups connected with silanol groups via water molecules. During heating, these bonds are destroyed, and amino groups are connected to each

other via hydrogen bonds, as evidenced by the appearance of the bands at 1580 cm^{-1}.[19] In addition, the removal of water, makes possible to identify two low-intensity absorption bands in the 3280-3370 cm^{-1} range related to $v_{s,as}$(NH) stretching of amino groups involved in hydrogen bonds. Finally, it should be mentioned that the presence of silanol groups in the surface layer of the sample ***N2*** is proved by the absorption band at ~3650 cm^{-1} (Fig. FS6).

The DRIFT spectra of the samples with amino/methyl groups (***NMh*** as example, Fig. FS6, spectrum 3) have sharp absorption band at 1273 cm^{-1}, which is absent in the DRIFT spectra of other samples and can be attributed to $\delta_s(CH_3)$ of methyl group bound to a silicon atom. At the 1415 cm^{-1} in the DRIFT spectra of these samples the band of low intensity is observed. This band relates to the asymmetric bending of methyl groups $\delta_{as}(CH_3)$.

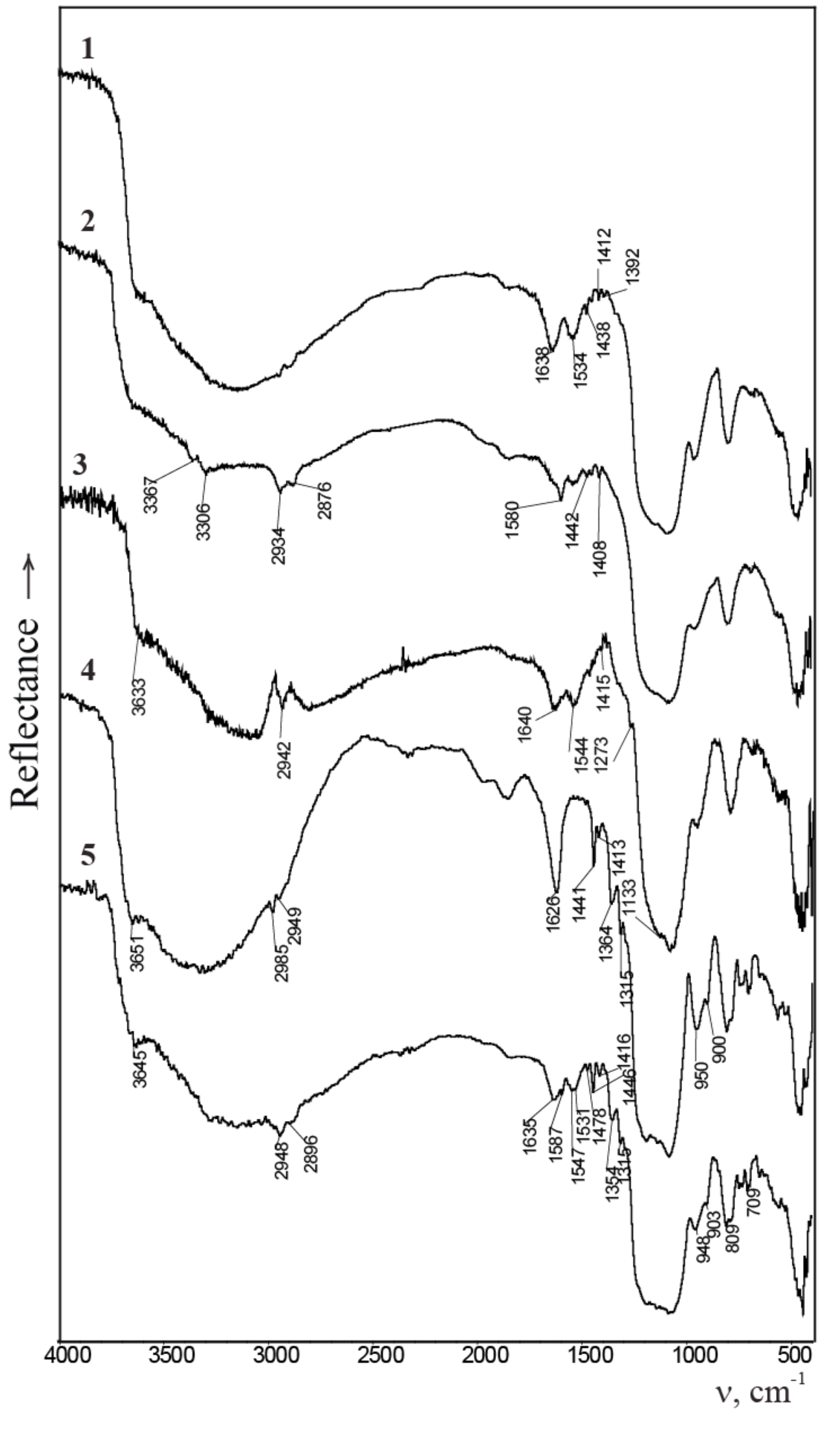

Figure S6: DRIFT spectra of samples: 1– ***N2*** (20°C), 2 – ***N2*** (100°C), 3 – ***NMh*** (20°C), 4 – ***F2***, 5 – ***NF3***.

The presence of perfluorooctyl groups in monofunctional fluorine-containing samples (sample ***F2*** in Fig. FS6) was confirmed by a band of medium intensity with a frequency of ~1315 cm^{-1} corresponding to $\nu_{as}(CF)$.[4] This absorption band is not observed in samples not containing a fluoroalkyl residue. However, the symmetric stretching band $\nu_s(CF)$ somewhat overlaps with stretching vibrations of polysiloxane network (broad intense adsorption band of siloxane bonds, ≡SiOSi≡, in the range 1000-1200 cm^{-1}), so it is difficult to identify it clearly. The signal (shoulder) at ~900 cm^{-1}, overlapping with a broad medium intensity band of δ(Si-OH) vibrations at 950 cm,$^{-1}$ also indicates the presence of CF_3 groups [4, 5].Indirectly, the presence of $≡Si(CH_2)_2(CF_2)_5CF_3$ groups in samples is testified by absorption bands at ~1364, ~1413 cm^{-1} (weak), and ~1441 cm^{-1} in their IR spectra (Fig. FS6), which can be attributed to $\omega(CH_2)$, $\delta(Si–CH_2)$, and $\delta_{as}(CH_2)$ respectively. Absorption bands characteristic of symmetric and asymmetric stretching vibrations of C-H bonds are also present in the region 2900-2985 cm^{-1}, but they overlap with broad absorption band of ν(OH) of adsorbed water at ~ 3000-3400 cm^{-1}.

The DRIFT spectra of bifunctional samples with fluorine and amine containing groups in the surface layer revealed adsorption bands characteristic of both amino- and perfluorooctyl functional groups mentioned above, thus witnessing their incorporation in the samples (Fig. FS6). For example, the spectra of samples ***NF3*** revealed an absorption band at 1547 cm^{-1}, which refers to the $\delta(NH_2)$ bending vibrations of amino groups. Upon heating the sample ***NF3*** to 100°C the $\delta(H_2O)$ bending band of water molecules at 1640 cm^{-1} vanishes from its IR spectrum, and the $\delta(NH_2)$ band of amino groups shifts to 1590 cm^{-1}. The removal of water at 100°C allows identification of absorption bands in the

3260-3370 cm^{-1} infrared spectrum region, belonging to $\nu_{s,as}$(NH) stretching vibrations of amino groups involved in the hydrogen bonds. Compared with the amine samples (***N2*** and ***NMh***), the IR spectra of fluorinated samples (***F2*** and ***NF3***, Fig. FS6) have easier identifiable absorption band at ~ 3650 cm^{-1}, which undoubtedly belongs to the silanol groups.

Nitrogen adsorption studies

Table TS1 presents specific surface areas for some samples calculated from low-temperature nitrogen adsorption isotherms. These data are consistent with the SEM data. Thus, S_{sp} of all amino samples is 10-43 m^2/g, which is due to rather large size of their particles (about 280-720 nm). In addition, an increase in the synthesis temperature causes an increase in the particles' diameter and, as a consequence, a decrease in the S_{sp}. (samples ***N4*** and ***N4h***). Only sample ***N4i*** has diameter of submicroparticles 140 nm, but they agglomerate each other. Bifunctional samples (Table TS1) with amino/methyl groups have developed porous structure. Apparently the particles consist of smaller particles packed in a certain order. The relatively high values of S_{sp} are observed for some samples with fluorine-containing groups (***F2***, ***FN1***, Table TS1). This is an indirect confirmation that their SEM images are likely to present the secondary structures, but clearly it can be argued only for sample ***F2*** (Fig. FS3). For this sample it is consistent with structural adsorption analysis.

TGA studies

Thermal analysis data indicate the presence of functional groups and water in the synthesized samples. Thus, Fig. FS7 presents thermograms for samples ***N4h***, ***NMh***, ***F2*** and ***NF4***. All thermograms are characterized by weight loss in the temperature range of 90-110°C, which can be associated with the removal of water and residual solvent. The results of the thermal analysis showed that the samples with monofunctional fluorine-containing layer are the most stable. Their thermal destruction starts above 400°C (sample ***F2*** in Fig. FS7). The processes of destruction of their organic layer are similar to the xerogels synthesized at the same TEOS:PFES ratios [6]. The DTG curves for bifunctional samples contain a peak at lower temperatures about 290°C (***FN4*** and ***NMh*** in Fig. FS7) associated with the removal of surface amino groups. The decomposition of amino groups in pure amino sample (***N4h***) starts at slightly lower temperature (270°C, Fig. FS7) and is consistent with the data for xerogels containing 3-aminopropyl groups [7, 8].

According to Table TS1, amino groups content in spherical silica particles is in the range 0.5-2.0 mmol/g (at the ratio of TEOS:APTES=3:1), which is about 2 times less than expected from the ratio of reactive alkoxysilanes. The data in this table suggest that several factors determine amino groups content: the components ratio (samples ***N1*** and ***N2***), the order of alkoxysilanes introduction in the reaction solution (samples ***N2*** and ***N3***), the synthesis temperature (samples ***N4i***, ***N4***, and ***N4h***); little effect is produced by the amount of used ammonia (samples ***N2*** and ***N4***) [9]. The accessibility and hydrolytic stability of amino groups is also of importance. The results of elementary microanalysis of the amounts of amino groups for the samples ***N4i***, ***N4***, and ***N4h*** and their TGA

are quite similar and close to the theoretical. The data obtained for these samples by acid-base titration are however revealing 1.5 times lower values indicating either instability of the surface layer or poor accessibility of the groups for the protonation. For the bifunctional samples we observed the amount of functional groups 1.8-2.0 mmol/g at twice lower amount of APTES. Introduction of the methyl groups is thus either stabilizing the surface layer or leading to enhanced accessibility of amino groups (the latter correlates well with the data from the adsorption of Cu^{2+} cations, please, see below).

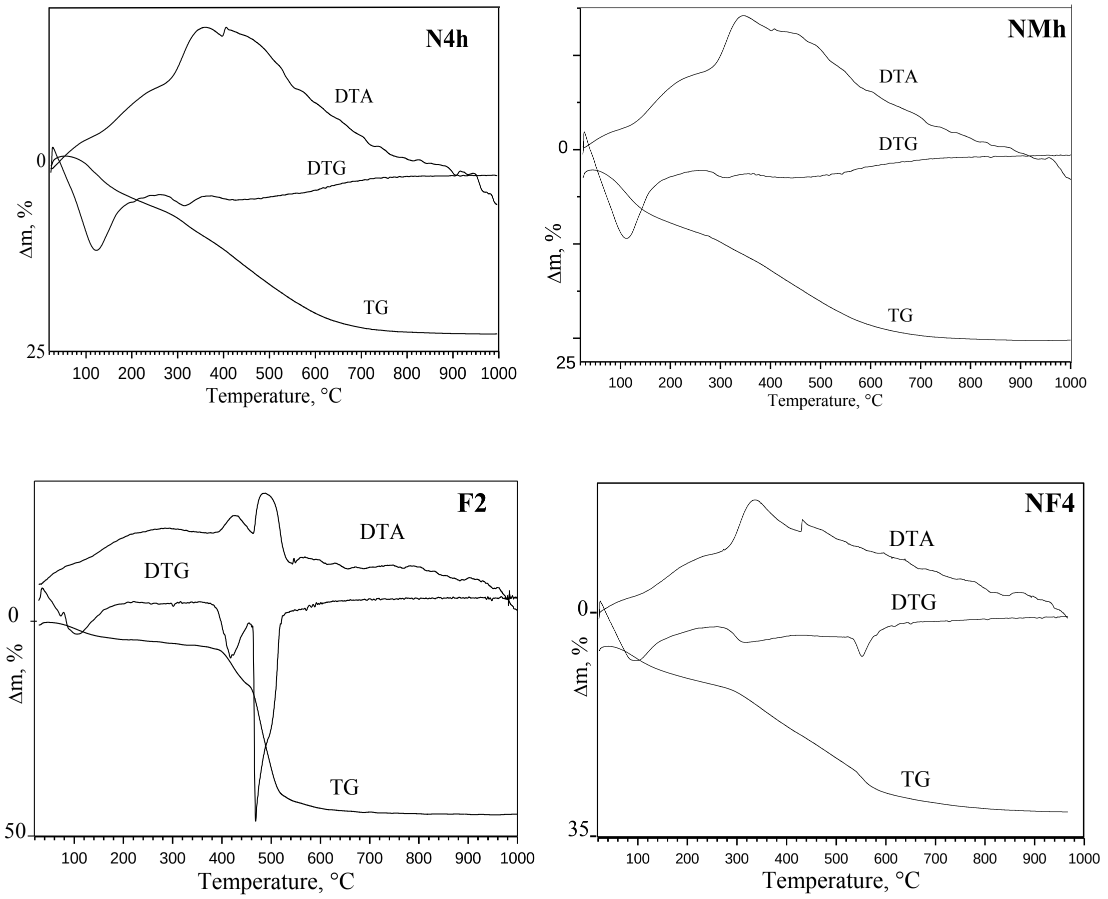

Figure S7: Thermograms for the samples with mono- and bifunctional surface layers.

Composition of the surface complexes with monofunctional layers

For monofunctional ***N4*** type samples synthesized at different temperatures, the compositions of the Cu^{2+}:Lig complexes are also different. Complexes Cu^{2+}:Lig= 1:2 form during copper(II) ions sorption by the sample obtained at room temperature; whereas, the number of ligands in the coordination sphere of copper is higher for samples obtained at lower (***N4i***) and higher (***N4h***) temperatures. In other words, there are several factors that influence the composition of the surface complexes, and this effect may be contradictory.

The types of copper(II) adsorption isotherms for the samples also confirm the above-mentioned observation (Fig. 6).

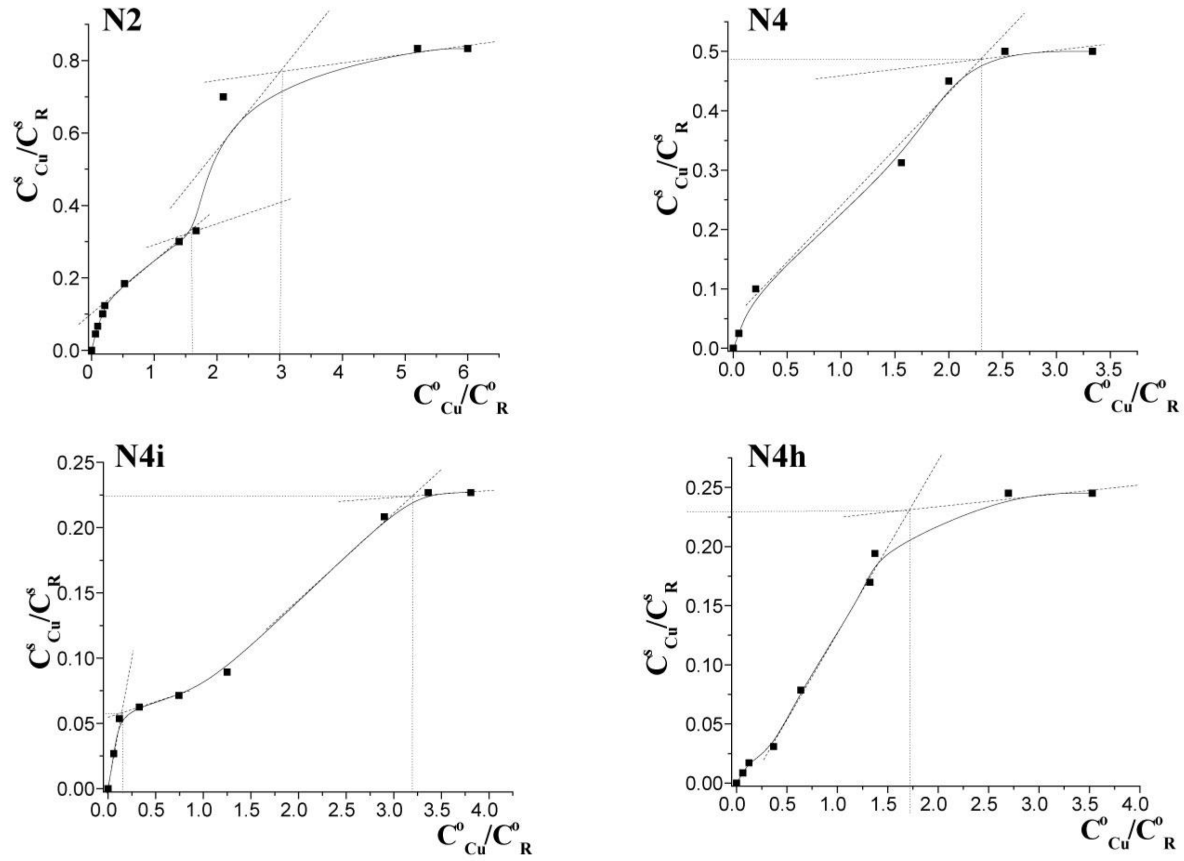

Figure S8: Adsorption isotherms for determination of the metal ligand ratios in the copper(II) complexes with amino groups in monofunctional layers.

Whereas simple for majority of samples, the copper(II) adsorption isotherms of samples ***N4i*** and ***N2*** have clear bends (Fig. 6, FS8). If for the first sample such bend is observed at a low C^0_{Cu}:C^0_R ratio and in a wide range (see Fig. 7), for the later sample it is abrupt at 1.5. It is worth noting that the synthesis of sample ***N4i*** was conducted at low temperature, while monofunctional sample ***N2*** was obtained at room temperature and using different synthesis technique. Obviously, in both cases there is different composition of copper(II) complexes formed in the surface layer of the samples.

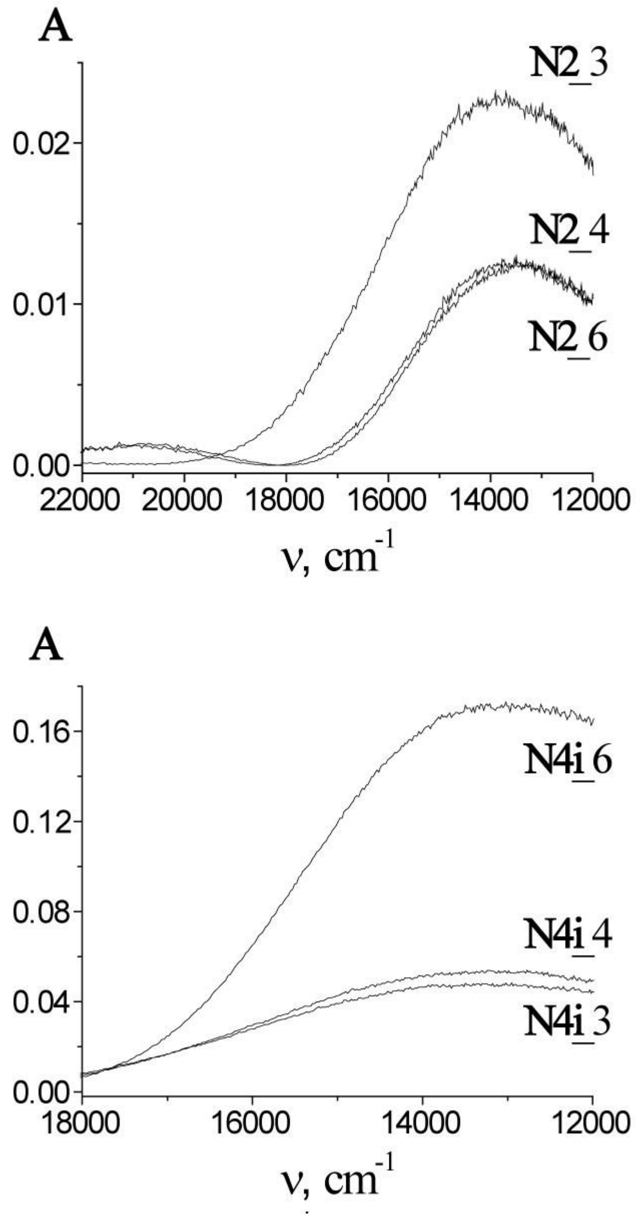

Figure S9: The EDSR spectra of the copper(II) complexes with monofunctional layers.

Adsorption of organic molecules on the monofunctional perfluoroalkyl functionalized layers

In the case of acetonitrile vapor adsorption isotherm for sample ***F2***, at low fillings it coincides with *n*-hexane adsorption isotherm. But with increasing P/Ps, acetonitrile adsorption curve is going higher, which may result from different

molecular sizes of acetonitrile and *n*-hexane. Water adsorption isotherm curve is lower, confirming the hydrophobicity of the sample.

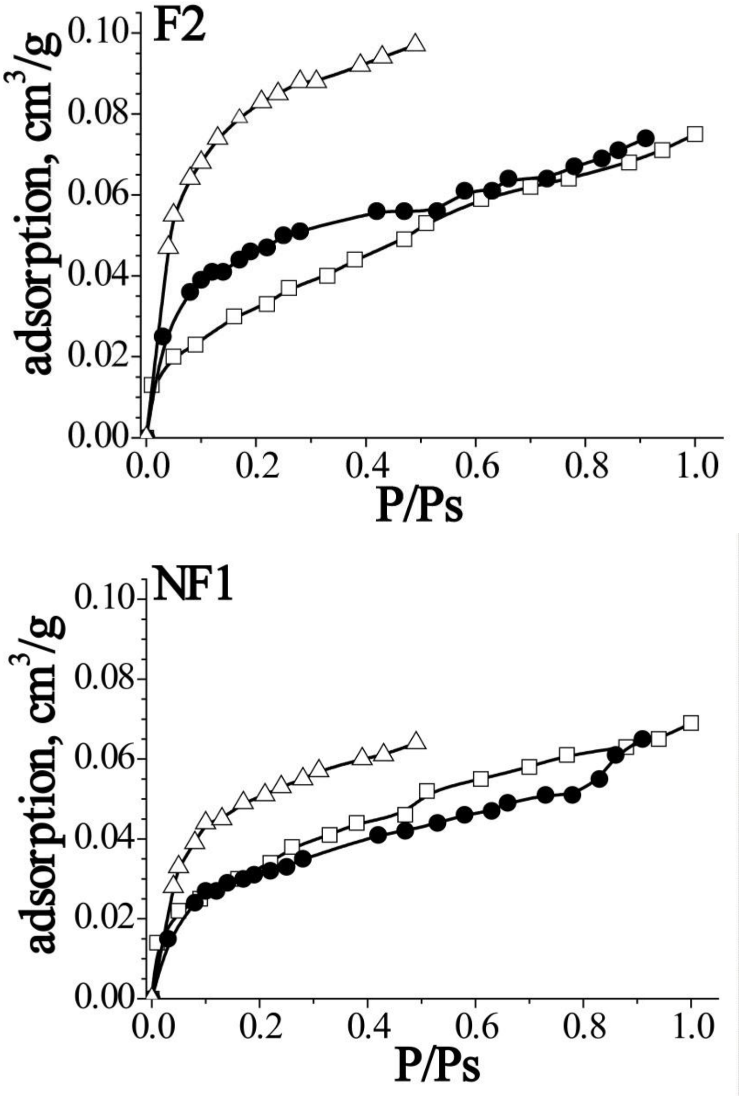

Figure S10: Adsorption isotherm of *n*-hexane (●), water (□) and acetonitrile (Δ) vapors.

Table S1: Some conditions for the syntheses and properties of nanoparticles.

Samples	TEOS/ trifunctional silanes ratio	Particles *d*, nm	S_{sp}, m^2/g	[a]$C_{c.g.}$, mmol/g	[b]Δm, %	SSC, mmol/g (Cu^{2+})
$SiO_2/{\equiv}Si(CH_2)_3NH_2$ ***N1***	1/1	300	11	2.7	15.9	1.3
$SiO_2/{\equiv}Si(CH_2)_3NH_2$ ***N2***	3/1	360	14	1.9	11.2	0.8
$SiO_2/{\equiv}Si(CH_2)_3NH_2$ ***N3***	3/1	460	10	2.1	12.3	0.1
$SiO_2/{\equiv}Si(CH_2)_3NH_2$ ***N4***	3/1	280	43	2.6	15.3	1.0
$SiO_2/{\equiv}Si(CH_2)_3NH_2$ ***N4i***	3/1	140	17	2.4	14.2	0.3
$SiO_2/{\equiv}Si(CH_2)_3NH_2$ ***N4h***	3/1	720	15	2.9	17.0	0.4
$SiO_2/{\equiv}Si(CH_2)_3NH_2/{\equiv}SiCH_3$ ***NM***	3/0.5/0.5	180	132	–	11.6	0.7
$SiO_2/{\equiv}Si(CH_2)_3NH_2/{\equiv}SiCH_3$ ***NMi***	3/0.5/0.5	160	208	–	-	0.8
$SiO_2/{\equiv}Si(CH_2)_3NH_2/{\equiv}SiCH_3$ ***NMh***	3/0.5/0.5	120	164	–	14.6	1.9
$SiO_2/{\equiv}Si(CH_2)_2(CF_2)_5CF_3$ ***F1***	3/1	270	40	1.8	64.1	–
$SiO_2/{\equiv}Si(CH_2)_2(CF_2)_5CF_3$ ***F2***	3/0.5	180	160	1.4	47.4	-
$SiO_2/{\equiv}Si(CH_2)_3NH_2/{\equiv}Si(CH_2)_2(CF_2)_5CF_3$ ***NF1***	3/0.25/0.25	210	110	–	31.5	0.49
$SiO_2/{\equiv}Si(CH_2)_3NH_2/{\equiv}Si(CH_2)_2(CF_2)_5CF_3$ ***NF2***	3/0.5/0.1	230	40	–	20.7	0.8
$SiO_2/{\equiv}Si(CH_2)_3NH_2/{\equiv}Si(CH_2)_2(CF_2)_5CF_3$ ***NF3***	3/0.5/0.5	180	50	–	45.7	0.60
$SiO_2/{\equiv}Si(CH_2)_3NH_2/{\equiv}Si(CH_2)_2(CF_2)_5CF_3$ ***NF4***	3/1/0.1	190	13	–	25.6	1.24

[a]The content of functional groups calculated assuming TGA analysis for monofunctional samples

[b]Δm after water removal (150–200°C)

Table S2: ^{13}C CP/MAS NMR spectra signal reference.

Signal reference	Chemical shift (ppm)			
	N2	***N4h***	***NMh***	***NF3***
$\equiv Si\text{-}\underline{C}H_2\text{-}CH_2\text{-}CH_2\text{-}N$	10.6	10.5	10.5	10.9
$\equiv Si\text{-}CH_2\text{-}\underline{C}H_2\text{-}CH_2\text{-}N$	22.5	22.4	22.4	25.4
$\equiv Si\text{-}CH_2\text{-}CH_2\text{-}\underline{C}H_2\text{-}N$	43.8	43.0	43.4	43.8
$\equiv Si\text{-}\underline{C}H_3$	-	-	−2.9	-
$\equiv Si\text{-}\underline{C}H_2\text{-}CH_2\text{-}(CF_2)_5\text{-}CF_3$	-	-	-	3.8
$\equiv Si\text{-}CH_2\text{-}\underline{C}H_2\text{-}(CF_2)_5\text{-}CF_3$	-	-	-	65.2
$\equiv Si\text{-}(CH_2)_2\text{-}\underline{C}F_2\text{-}\underline{C}F_2\text{-}\underline{C}F_2\text{-}\underline{C}F_2\text{-}CF_2\text{-}CF_3$	-	-	-	110–113
$\equiv Si\text{-}(CH_2)_2\text{-}CF_2\text{-}CF_2\text{-}CF_2\text{-}CF_2\text{-}\underline{C}F_2\text{-}CF_3$	-	-	-	108 (sh)
$\equiv Si\text{-}(CH_2)_2\text{-}(CF_2)_5\text{-}\underline{C}F_3$	-	-	-	119.4
$\equiv Si\text{-}O\text{-}\underline{C}H_2\text{-}CH_3$	19.0	19.0 (sh)	19.5 (sh)	-
$\equiv Si\text{-}O\text{-}CH_2\text{-}\underline{C}H_3$	59.6	58.3	-	-

Table S3: Signals attribution in ^{13}C CP/MAS NMR spectra of samples with amino groups.

Signal	Chemical shift, ppm	
	N2	***N5***
≡Si-$\underline{C}H_2$-CH_2-CH_2-N	10.5	11.1
≡Si-O-$\underline{C}H_2$-CH_3	18.1	-
≡Si-CH_2-$\underline{C}H_2$-CH_2-N	22.4	22.6
≡Si-CH_2-CH_2-$\underline{C}H_2$-N	43.2	48.8
≡Si-$(CH_2)_3$-N-$\underline{C}H_2$-CH_2-N	-	48.8
≡Si-$(CH_2)_3$-N-CH_2-$\underline{C}H_2$-N	-	40.4
≡Si-O-$\underline{C}H_3$	-	51.0
≡Si-O-CH_2-$\underline{C}H_3$	58.7	-

Table S4: Elemental analysis data and concentrations of functional groups calculated from them.

Sample	N, mass %	C, mass %	H, mass %	Concentration of aminogroups, mmol/g			Concentration of C-containing groups, mmol/g			
				Theor. data	Elem. anal. data	Titration data	Methyl groups		C-Fluor groups	
							Theor .data	Elem. anal. data	Theor. data	Elem.anal. data
N1	3.57	9.40	3.37	5.8	2.6	2.0	-	-	-	-
N2	2.20	5.51	2.74	3.3	1.6	1.3	-	-	-	
N3	1.57	6.77	2.47	3.3	1.1	0.5		1.1[a]	-	
N4	3.77	10.06	3.35	3.3	2.7	2.0	-	0.1[a]	-	-
N4i	3.37	9.37	3.13	3.3	2.4	1.4	-	0.2[a]	-	-
N4h	4.69	11.28	3.82	3.3	3.4	1.7	-	-	-	-
NM	3.35	8.32	3.55	1.9	2.4	2.0	1.9	0.1	-	-
NMi	3.00	8.44	3.05	1.9	2.1	1.8	1.9	0.6	-	-
NMh	3.93	10.89	3.70	1.9	2.8	1.9	1.9	0.6	-	-
NF1	1.19	9.21	1.47	0.8	0.9	0.5	-	-	0.8	0.6 (1.0[b])
NF2	1.47	8.01	1.96	1.8	1.0	1.0	-	-	0.4	0.4 (0.5[b])
NF3	1.61	13.63	1.74	1.2	1.2	0.7	-	-	1.2	1.0 (1.2[b])
NF4	2.79	9.89	2.80	3.0	2.0	1.7	-	-	0.3	0.3 (0.4[b])

[a]residual ethoxy groups

[b]In brackets, there are given the content of fluor-containing groups calculated from EDXS analysis

Table S5: Kinetic sorption parameters obtained using pseudo-first and pseudo-second-order models for metals sorption

Sample	$C_{funct.gr.}$ mmol/g	pseudo-first-order		pseudo-second-order		
		k_1, min^{-1}	R^2	$a_{eqv,}$ mmol/g	k_2, g/mmol/min	R^2
N2	1.0	0.033±0.011	**0.957**	0.527±0.146	0.033±0.025	0.705
N4	2.0	0.089±0.013	0.995	0.639±0.010	0.350±0.139	**0.999**
N4i	1.43	0.059±0.016	0.869	0.137±0.009	0.435±0.211	**0.984**
N4h	1.7	0.029±0.003	0.977	0.185±0.009	0.264±0.094	**0.989**
NM	2.0	0.144±0.005	0.998	0.675±0.007	0.735±0.358	**0.999**
NMi	1.8	0.104±0.003	0.998	0.431±0.012	0.428±0.215	**0.996**
NMh	1.9	0.080±0.006	0.987	0.593±0.005	0.552±0.177	**0.999**
NF1	0.46	0.031±0.005	0.973	0.399±0.015	0.116±0.037	**0.996**
NF2	1.0	0.006±0.001	0.908	0.858±0.020	0.213±0.114	**0.998**
NF3	0.66	0.028±0.001	0.999	0.612±0.004	0.236±0.038	**0.999**
NF4	1.73	0.007±0.001	0.975	1.106±0.012	0.035±0.010	**0.999**

Table S6: Parameters of copper(II) adsorption using Langmuir and Freundlich isotherm models.

Sample	Me/Li ratio	Langmuir isotherm			Freundlich isotherm	
		$a_{max,}$ mmol/g	$K_{L,}$ L/mmol	R^2	K_F	R^2
N2	0.62	1.119	0.301	0.834	0.224	**0.972**
N4	0.5	1.472	0.223	0.937	0.222	**0.983**
N4i	0.21	0.415	0.346	0.778	0.107	**0.926**
N4h	0.24	1.108	0.082	0.489	0.078	**0.969**
NM	0.35	0.998	0.227	0.912	0.155	**0.951**
NMi	0.44	1.418	0.126	0.773	0.148	**0.976**
NMh	1.0	3.197	0.075	0.768	0.206	**0.959**
NF1	1.06	0.531	1.008	**0.997**	0,224	0.895
NF2	0.80	0.943	0.797	**0.989**	0.374	0.961
NF3	0.90	0.695	1.258	**0.989**	0.350	0.878
NF4	0.72	1.364	0.395	**0.998**	0.356	0.920

Table S7: Parameters of the EPR spectra of copper(II) complexes formed on the surface of some spherical carriers.

Sample	Ratio Metal/Lig in the surf. layer (C^S_{Cu}:C^S_L)	Ratio Metal/Lig in the solution (C^0_{Cu}:C^0_L)	g_{II}	$g_{\perp}$	$A_{II}\cdot10^{-4}$, cm^{-1}
NF4_3	1:5.2	1:4	2.25	2.045	157
NF4_4	1:2.7	1:2	2.24	2.048	164
NF4_6	1:0.87	1:0.5	2.26	2.046	165
N2_3	1:3.9	1:4	2.24	2.071	167
N2_4	1:2	1:2	2.25	2.053	164
N4i_3	1:4.3	1:4	2.27	2.044	150
N4i_4	1:2.1	1:2	2.27	2.045	150

References

1. Gordon, A.J.; Ford, R.A. *The Chemist' Companion*; Wiley: New York, 1972.

2. Lin-Vien, D.; Colthup, N.B.; Fateley, W.G.; Grasselli, J.G. *The Handbook of Infrared and Raman Characteristic Frequencies of Organic Molecules*; Academic Press: London, 1991.

3. Zaitsev, V. *Complexing silicas: preparation, structure of bonded layer, surface chemistry*; Folio: Kharkov, 1997 (in Russ.).

4. Reynolds, J.G.; Coronado, P.R;. Hrubesh, L.W. *J. Non-Cryst. Solids* **2001***, 292*, 127-137.

5. Reynolds, J.G.; Coronado, P.R.; Hrubesh, L.W. *Energy Sources* **2001***, 23*, 831-834.

6. Bagwe, R.P.; Hilliard, L.R.; Tan, W.H. *Langmuir* **2006,** *22,* 4357-4362.

7. Zub, Yu.; Chuiko, A. Salient Features of Synthesis and Structure of Surface of Functionalized Polysiloxane Xerogels. In *Colloidal Silica: Fundamentals and Applications;* Bergna, H.; Roberts, W., Eds.; Surfactant science series, Vol.131; CRC Press: Boca Raton, 2006; pp 397-424.

8. Zub, Yu. Design of functionalized polysiloxane adsorbents and their environmental applications. In *Sol-Gel Methods for Materials Processing;* Innocenzi, P.; Zub, Yu.; Kessler, V., Eds.; Springer: Dordrecht, 2008; pp 1-29.

9. Melnyk I.V.; Zub, Yu. L. *Micropor. Mesopor. Mater.* **2012**, *154*, 196-199.
